# USP30-mediated Deubiquitination of Hexokinase 2 controls the metabolic fate of glucose and tumor progression

**DOI:** 10.1038/s41419-026-08459-w

**Published:** 2026-02-14

**Authors:** Zhang Haowei, Xiaolin Li, Weijie Liao, Xuan Qin, Yapei Jiang, Haitao Yang, Hongli Zeng, Yuetong Li, Weidong Xie, Yaou Zhang, Naihan Xu

**Affiliations:** 1 School of Food and Drug, Shenzhen Polytechnic University, Shenzhen, China; 2 State Key Laboratory of Chemical Oncogenomics, Institute of Biopharmaceutical and Health Engineering, Tsinghua Shenzhen International Graduate School, Tsinghua University, Shenzhen, China; 3 Department of Hematology and Oncology, International Cancer Center, Shenzhen University General Hospital, Shenzhen University, Shenzhen, China; 4 Fuwai Shenzhen Hospital, Chinese Academy of Medical Sciences, Shenzhen, China

**Keywords:** Cancer metabolism, Ubiquitylation, Ubiquitylation

## Abstract

The mitochondria-localized deubiquitinase USP30 regulates mitophagy and mitochondrial homeostasis, playing a significant role in the progression of neurodegenerative diseases. However, its role in cancer remains poorly understood. Through metabolism-related gene set enrichment analysis, we identified USP30 as a key modulator of glucose metabolism in cancer cells. Utilizing quantitative proteomic and ubiquitinomic approaches coupled with co-immunoprecipitation assays, we elucidated that USP30 regulates glycolysis by interacting with hexokinase HK1 and HK2, a process dependent on its enzymatic activity. USP30 modulates the ubiquitination profile of HK1 and HK2 by preferentially removing atypical ubiquitin chains, thereby enhancing their stability, mitochondrial localization, VDAC1 binding and hexokinase activity. Lysine 144 emerges as a critical regulatory site for USP30-mediated deubiquitination of HK2. Mutation of K144 enhances HK2 stability, increases its mitochondrial localization and binding to VDAC1, and significantly augments hexokinase activity. Furthermore, the HK2 K144 mutation markedly enhances tumor cell glycolysis, fostering increased proliferation and migration both in vitro and in vivo. These findings underscore USP30 as a novel regulator of glycolysis in cancer cells via modulation of HK2 ubiquitination dynamics, suggesting its potential as a therapeutic target in cancer metabolism.

## Introduction

Elevated glucose metabolism is a defining feature of tumor cells, supporting their growth and metastasis through increased glucose consumption and lactate production. This phenomenon, known as the Warburg effect or aerobic glycolysis, plays a crucial role in tumor cell metabolic reprogramming [1, 2]. Aerobic glycolysis enables tumor cells to adapt to stressful conditions, sustaining rapid proliferation, migration, invasion, and metastasis. Additionally, it provides essential precursors for biosynthetic pathways, suppresses antitumor immune responses, and participates in cell signaling mechanisms involving reactive oxygen species [3, 4].

Hexokinase is the first rate-limiting enzyme in glycolysis, catalyzing the conversion of glucose to glucose-6-phosphate (G6P). This irreversible catalytic reaction plays a crucial role in cellular metabolism and enhances glucose uptake in tumor cells [3, 5, 6]. G6P generated by hexokinase serves as a pivotal metabolite intersecting glycolysis, the pentose phosphate pathway, the glucosamine pathway, and glycogenesis, influencing numerous synthetic metabolic pathways crucial for cell growth and proliferation [4]. In mammals, the hexokinase family comprises five members: HK1, HK2, HK3, GCK (glucokinase), and HKDC1 (hexokinase domain containing 1), each encoded by distinct genes [7, 8]. Notably, HK1, HK2, and HKDC1 possess mitochondrial targeting sequences, allowing them to localize to the outer mitochondrial membrane (OMM). HK1 and HK2 specifically bind to the OMM and interact with the voltage-dependent anion channel (VDAC). These OMM-bound hexokinases (mtHKs) are positioned near intra-mitochondrial ATP, thereby promoting glycolysis. G6P can allosterically inhibit HK1 and HK2, leading to their dissociation from mitochondria [7, 9–11]. Beyond their roles in glucose metabolism, hexokinases also display “moonlighting” functions, participating in cell death, immune responses, and various cellular processes [6, 9, 12, 13].

Ubiquitination, a widespread post-translational modification (PTM), plays a crucial role in regulating various signaling pathways. The balance between ubiquitination and deubiquitination, mediated by E3 ubiquitin ligases and deubiquitinating enzymes (DUBs), is essential for maintaining proteostasis and responding to cellular stimuli. DUBs significantly influence the hallmark features of cancer, including metabolic reprogramming, sustained proliferation, invasion, and metastasis [14]. They achieve this by modulating key substrates in signaling pathways, transcription factors, and metabolic enzymes, thereby impacting glucose metabolism and tumorigenesis. USP30, a mitochondrial DUB located on the OMM, is known for maintaining mitochondrial homeostasis and inhibiting mitophagy. It has also been implicated in neurodegenerative diseases [15–20]. While the physiological roles of USP30 in mitochondrial homeostasis and neurodegenerative diseases are well-established, its involvement in tumorigenesis and progression remains less understood. Recent findings suggest that USP30 contributes to lipid metabolism by stabilizing DRP1 and ACLY, promoting tumorigenesis and progression in hepatocellular carcinoma [21, 22]. USP30 has also been shown to facilitate breast cancer progression by stabilizing Snail [23].

In this study, we identified USP30 as a key regulator of glucose metabolism in cancer cells. USP30 modulates glycolysis through its interactions with hexokinase 1 (HK1) and hexokinase 2 (HK2). Specifically, USP30 selectively removes atypical ubiquitin chains from these hexokinases, enhancing their stability, mitochondrial localization, and enzymatic activity. Our analyses revealed that lysine 144 of HK2 serves as a critical regulatory site for USP30-mediated deubiquitination. The introduction of a K144R mutation in HK2 not only stabilizes the protein but also promotes its mitochondrial localization, resulting in a significant increase in hexokinase activity. Furthermore, the HK2 K144 mutation markedly enhances glycolysis in tumor cells, driving increased proliferation and migration both in vitro and in vivo. These findings establish USP30 as a novel regulator of glycolysis in cancer cells through its modulation of HK2 ubiquitination dynamics, highlighting the potential of targeting USP30 as a therapeutic strategy for manipulating cancer metabolism.

## Materials and methods

### Cell culture and transfection

The cell lines HeLa, HepG2, and HCT116 were obtained from ATCC, while HEK293 and HEK293T cell lines were sourced from the Cell Bank at the Shanghai Institute of Biochemistry and Cell Biology (SIBCB), Chinese Academy of Sciences. HeLa, HepG2, HEK293, and HEK293T cells were cultured in DMEM (Gibco, C11995500BT), whereas HCT116 cells were maintained in McCoy’s 5A medium. All culture media were supplemented with 10% fetal bovine serum (FBS) (ExCell, FSP500) and 1% penicillin-streptomycin (PS) (Sangon Biotech, E607011). Cells were grown in a humidified incubator at 37 °C with 5% CO_2_. Cell lines used in this study were not listed in the ICLAC database, and no mycoplasma contamination was detected, as confirmed by the Mycoplasma qPCR Detection Kit (Beyotime, C0303S). Transfection of siRNA or plasmid DNA were carried out using the jetPRIME transfection reagent (Polyplus, 101000046) following the manufacturer’s instructions, with a final siRNA concentration of 50 nM.

### Plasmid construction

Full-length complementary DNAs (cDNAs) of human glucose metabolic enzymes and USP30 were amplified by PCR using PrimeSTAR Max DNA polymerase (Takara, R040) with primers containing appropriate restriction sites. These genes were then cloned into pVAX1-3xFlag-C, pcDNA3.1(+)-His/HA, or pEGFP-N1 vectors for transient expression. For lentiviral overexpression studies, constructs were created using the pCDH-CMV-MCS-Neo vector. Mutations and truncations of HK1, HK2, and USP30 were introduced using the Mut Express Fast Mutagenesis Kit (Vazyme, C214/C215).

Gene knockout was achieved using the CRISPR/Cas9 method. Short guide RNAs (sgRNAs) targeting upstream exons of HK2 and USP30 were designed with the CRISPR design tool (http://crispr.mit.edu/). For gene knockdown, short hairpin RNAs (shRNAs) targeting HK2 and USP30 transcripts were designed based on established guidelines (see Supplementary Table S1). Annealed gRNA oligos were inserted into the linearized LentiCRISPRv2 vector (Addgene, #52961) digested with BsmBI (NEB #R0580) or the PX459 vector (Addgene, #62988) digested with BbsI-HF® (NEB #R3539). Similarly, annealed shRNA oligos were cloned into the pLKO.1-TRC vector (Addgene, #10878) digested with AgeI (NEB, R3552S) and EcoRI (NEB, R3101S). The resulting plasmids were verified by restriction enzyme digestion and DNA sequencing (BGI).

### Lentivirus production and stable cell lines

Lentivirus packing was performed by co-transfecting HEK293T cells with the lentiviral packaging plasmid psPAX2 (Addgene, #12260), the envelope plasmid pCMV-VSV-G (Addgene, #8454), and either the LentiCRISPRv2-gRNAs or pLKO.1-shRNAs vectors. Six hours post-transfection, the media were replaced with fresh DMEM containing 10% FBS. Lentivirus-containing media were collected at 48- and 72-h post-transfection, filtered through a 0.45-µm filter, and then precipitated by adding a 5X concentration buffer (PEG-8000, 50% (W/V); 0.1 M NaCl in PBS). The mixture was incubated at 4 °C overnight, followed by centrifugation at 3500 × *g* for 30 min at 4 °C. The supernatant was carefully removed, and the pellet was resuspended in 1/100 of the original volume using DMEM.

For infection, cells at ~30% confluency in a 12-well plate were treated with lentivirus in the presence of 8 µg/mL polybrene (Sigma). After 72 h, selection for stable cell lines was performed with 1.5 µg/mL puromycin (Beyotime, ST551) for 5 days. To achieve non-constitutive expression of Cas9 and gRNAs, PX459-gRNAs was transfected into cells, and selection was carried out using 2 µg/mL puromycin for 3 days. Effectiveness of the knockout was confirmed by sequencing PCR products flanking the edited sites and by Western blotting.

For the re-expression of HK2 in HK2-KO or HK2-KD cells, lentivirus containing pCDH-CMV-MCS-Neo, pCDH-CMV-MCS-HK2(WT)-Neo, or pCDH-CMV-MCS-HK2(K144R)-Neo was packaged with psPAX2 and VSV-G. This lentivirus was used to infect HK2-KO/KD cell lines, with selection carried out using 500 µg/mL G418 (Gibco). All stable cell lines were validated by Western blotting.

### In vitro protein binding assay

HK2 and USP30 were cloned into pGEX-4T-1 (HK2-GST) and pET-28a (USP30-His) expression vectors, respectively. Recombinant proteins were expressed in *E. coli* through 0.1 mol IPTG induction overnight at 25 °C. HK2-GST and USP30-His proteins were purified using GST-Tag Protein Purification Kit (Beyotime, P2260S) with Magnetic Agarose Beads and His-Tag Protein Purification Kit (Beyotime, P2247S) with NTA-Ni Magnetic Agarose Beads according to the manufacturer’s protocols. In the GST pull-down assay, HK2-GST was immobilized on GST beads and then incubated with USP30-His at 4°C for 2 h with rotation. Conversely, in the His pull-down assay, USP30-His was bound to Ni-NTA magnetic beads prior to incubation with HK2-GST. Both assays were performed under native conditions, followed by three washes with the kit’s native wash buffer. GST- or His-tagged proteins alone were used as negative controls. Protein complexes were eluted by boiling in protein loading buffer at 100 °C for 10 min and were subsequently analyzed by Western blotting to detect protein-protein interactions.

### Immunoprecipitation and immunoblotting

For immunoprecipitation (IP), cells were washed twice with ice-cold PBS and then lysed using either IP buffer RIPA (Beyotime, P0013C) or NP-40 buffer (Beyotime, P0013F), supplemented with 1 mM PMSF (Beyotime, ST506) immediately before use. While indicated, protease inhibitor cocktails and PhosSTOP phosphatase inhibitor cocktails (Roche) were also added. Cell lysates were vortexed briefly and incubated on ice for 30 min, followed by centrifugation at 12,000 × *g* for 10 min at 4 °C. The resulting supernatants were used for subsequent experiments.

For endogenous protein co-immunoprecipitation, Protein G Dynabeads (Thermo, 10004D) were pre-incubated with the indicated antibody for 30 min at room temperature. The beads were washed twice with 0.02% PBST (Tween in PBS, V/V), and then the supernatants were added to the beads for overnight incubation at 4 °C with gentle rotation. For exogenous proteins with tags, anti-Flag pre-conjugated beads (Selleck, B26102) or anti-HA pre-conjugated beads (Selleck, B26202) were used, incubating with supernatants overnight at 4 °C. Following bead washing with PBST or IP buffer, bound proteins were eluted with SDS-PAGE loading buffer (Beyotime, P0285) and boiled for 7 min at 100 °C. The protein samples were then analyzed using Western blotting. SDS-PAGE was performed on a gel, followed by transfer to a nitrocellulose membrane (PALL, #66485). The membrane was blocked with 5% nonfat milk in TBS (25 mM Tris, 150 mM NaCl, 1% Tween) and then incubated with primary antibodies: anti-HK2 (Abcam, ab209847; WB, 1:2000), anti-HK1 (Abcam, ab150423; WB, 1:2000), anti-USP30 (Abcam, ab314749; WB, 1:500), anti-Flag (Proteintech, 20543-1-AP; WB, 1:2500), anti-USP30 (Santa Cruz Bio, sc-515235; IP), anti-VDAC1 (CST, #4661; WB, 1:1000), anti-Actin (Proteintech, 66009-1-Ig; WB, 1:2000), anti-Tubulin (Proteintech, 11224-1-AP; WB, 1:2000), and anti-GAPDH (Proteintech, 10494-1-AP; WB, 1:5000). Afterward, the membrane was probed with Horseradish peroxidase (HRP)-conjugated secondary antibodies, and protein signals were detected using an enhanced chemiluminescence (ECL) method.

### Ubiquitination assay

The prediction of ubiquitination sites of HK2 was performed in BDM-PUB website (http://bdmpub.biocuckoo.org/prediction.php). The lysine (K) sites with potential deubiquitination by USP30 were mutated to arginine (R) for subsequent assays. HEK293 cells were co-transfected with the indicated plasmids and incubated for 48 h. Before harvesting, cells were treated with 10 µg/mL MG132 (MCE, HY-13259) for 4 h to inhibit proteasomal degradation. Cells were lysed using RIPA buffer, and the cell lysates were incubated overnight with anti-Flag beads, with gentle rotation. After incubation, the beads were washed, and the proteins were subjected to immunoblotting (IB) using an anti-Ubiquitin antibody (CST, #3936).

### Measurements of ECAR and OCR

ECAR and OCR were measured using the Seahorse XFp Extracellular Flux Analyzer (Agilent-Seahorse) following the manufacturer’s protocol. Cells were seeded into 8-well miniplates (Agilent-Seahorse) in 100 µL of complete media per well and incubated at 37 °C overnight in a 5% CO₂ incubator. Simultaneously, a sensor cartridge was hydrated overnight at 37 °C in a non-CO₂ incubator using Agilent Seahorse XF Calibrant (Agilent). Prior to measurement, ECAR buffer (Agilent XF DMEM or RPMI 1640 supplemented with 2 mM L-Glutamine, pH 7.4) or OCR buffer (Agilent XF DMEM or RPMI 1640 supplemented with 10 mM Glucose, 1 mM Pyruvate, 2 mM L-Glutamine, pH 7.4) was prepared. Cells were washed twice with the respective buffer and then incubated in a non-CO₂ incubator at 37 °C for 1 hour. For ECAR measurements, cells were sequentially treated with 10 mM Glucose, 1.5 µM Oligomycin, and 50 mM 2-DG. For OCR experiments, cells were sequentially treated with 1.5 µM Oligomycin, 1.5 µM FCCP, and 1 µM Rotenone/Antimycin A (1:1). The ECAR and OCR data were analyzed using WAVE software (Agilent).

### Lactate production and glucose consumption assays

Cells were seeded in 12-well plate in triplicate and allowed to attach overnight. The media were then replaced with fresh serum-free media. The cells were incubated for a further 16 h or 4 h for measurement of glucose consumption or lactate production, respectively. The cultured media were collected for measurement of glucose and lactate concentrations. Lactate levels were determined using Micro Lactate Assay Kit (Abbkine, KTB1100). Glucose content was determined using Glucose Assay Kit (Beyotime, S0201). Glucose consumption was defined by the difference in glucose concentration in medium with or without cell incubation. Cells were collected and counted, and glucose consumption and lactate production were normalized with control group.

### Hexokinase activity assay

Hexokinase activity was assessed as described previously [24, 25]. Briefly, HEK293 cells transfected with the indicated plasmids were harvested after 48 h. Cells were harvested and resuspended in 0.5 mL of HK-Act lysis buffer (20 mM Tris, 150 mM NaCl, 1% Triton X-100, 5 mM EDTA, 10% glycerol). The cell lysates were homogenized at 4 °C by pipetting and then centrifuged at 12,000 × *g* for 15 min at 4 °C. The supernatants were collected for hexokinase assay and immunoblotting. For the assay, 10 µL of supernatants were added to 190 µL of reaction buffer in a 96-well plate, using the Hexokinase Activity Assay Kit (Solarbio, BC0745), in triplicate. Hexokinase activity was measured at A340 using a spectrophotometer, with optical density recorded every 30 s for 20 cycles to reflect NADPH concentration changes. The total hexokinase activity was calculated from the slope of the curve during the log phase. Hexokinase activities of different samples within the same experiment were normalized to the protein content in the lysate.

### Immunofluorescence

Cells cultured on glass coverslips in 12-well plates were fixed with 100% cold methanol on ice for 10 min. Following fixation, cells were permeabilized with 0.2% Triton X-100 in phosphate-buffered saline (PBS) for 10 min at room temperature. After permeabilization, the coverslips were washed twice with washing buffer (0.02% Triton X-100, 1.5% BSA, and 1 mM NaN3 in PBS). Blocking was performed with blocking solution (PBS containing 5% BSA, 0.02% Triton X-100, and 1 mM NaN3) for 1 h. The cells were then incubated with anti-HK2 (Abcam, ab209847; IF, 1:200), anti-HK1 (Abcam, ab150423; IF, 1:200), diluted in blocking solution, for 1 h Following primary antibody incubation, the coverslips were washed four times (5 min each) with washing buffer. The secondary antibodies, conjugated with Alexa Fluor 488, 594, or 633 (Invitrogen), were applied in blocking solution for 1 h at 37 °C in the dark. After secondary antibody incubation, the cells were stained with 1 µg/mL 4’,6-diamidino-2-phenylindole (DAPI) in washing buffer for 2 min, washed four times, and mounted with 90% glycerol before sealing with nail polish. Images were acquired using a Zeiss Laser Scanning Microscope.

### Isolation of mitochondria fraction

Mitochondria were isolated using the Cell Mitochondria Isolation Kit (Beyotime, C3601). Briefly, 2 × 10^7^ cells were harvested in 1 mL of fraction buffer containing 1 mM PMSF. A 50 µL aliquot of this cell suspension was mixed with 100 µL of RIPA buffer for whole cell lysate (WCL) preparation. The remaining cell suspension was homogenized 50–80 times using a Dounce glass homogenizer. The homogenates were then centrifuged at 1000 × *g* for 10 min at 4 °C. The supernatant was collected and further centrifuged at 10,000 × *g* for 10 min at 4 °C. The resulting supernatant and pellet were designated as the cytosolic (Cyto) and mitochondrial (Mito) fractions, respectively. Both mitochondrial pellets and cytosolic fractions were subsequently lysed with RIPA buffer and subjected to immunoblotting.

### Bioinformatics analysis

Gene expression data for cervical cancer (TCGA-CESC), liver Cancer (TCGA-LIHC) and colorectal cancer (TCGA-COAD) were obtained from GDC TCGA data portal (https://portal.gdc.cancer.gov/). Single-sample gene set enrichment analysis (ssGSEA) was performed using the GSVA v1.46.0 package in R (https://www.r-project.org/) with method = ‘ssgsea’. Two previously reported gene signatures were applied: “Lipid/Fatty Acid-Enriched (top 200 genes, cluster 1)” and “Nucleotide/Carbohydrate-Enriched (top 200 genes, cluster 2)” [26], from which the ssGSEA enrichment scores for each signature were derived.

### Cell proliferation assays

Cells were seeded in a 96-well plate at a density of 1 × 10^3^ cells per well (*n* = 5 per group) and incubated overnight in complete media. The media were then replaced with 100 µL of fresh serum-free media containing CCK-8 reagent (MCE, HY-K0301). After 1.5 h of incubation, the optical density was measured at 570 nm using a spectrophotometer, with background subtraction performed using a blank control. For the colony formation assay, cells were seeded in 6-well plate at 150 cells per well (*n* = 3 per condition). The media were refreshed every two days with complete media. After two weeks, cells were fixed with methanol for 15 min and stained with 0.1% crystal violet for 10 min. The stained colonies were thoroughly washed with PBS and imaged using the iBright Imaging System (Thermo, CL750).

### Transwell assay

Cell migration and invasion assays were performed using Transwell chambers with 8 µm pore size filters. For migration assays, the chambers were uncoated, while for invasion assays, they were coated with Matrigel. A cell suspension (3 × 10^4^ cells per well) was added to the upper chamber with serum-free medium, while the lower chamber contained complete medium with 10% FBS. cells were incubated for 36 h for migration assays and 48 h for invasion assays. Following incubation, cells on the bottom surface of the filters were fixed with methanol for 10 min at room temperature and then stained with 0.2% crystal violet. The number of migrated or invaded cells was quantified by counting five random microscopic fields from three independent replicates.

### Animal experiment

HepG2 cells from four experimental conditions, including control, HK2-KO, and HK2-KO reconstituted with either HK2 WT or K144R mutations, were cultured in DMEM with 10% FBS to 80–90% confluency in 10-cm plates. The cells were trypsinized, washed twice with ice-cold PBS, and resuspended in a 5:1 mixture of ice-cold PBS and Matrigel at a concentration of 4 × 10^7^ cells/mL. A 0.1 mL aliquot (4 × 10^6^ cells) was injected subcutaneously into the right or left flanks of male BALB/c nude mice (5 weeks old, *n* ≥ 6), obtained from Guangdong Medical Laboratory Animal Center. Tumor size was measured 2–3 times per week using calipers. Three weeks post-injection, tumors were collected, weighed, and their volumes were calculated using the formula: Volume = L × W² × 0.52, where L is length and W is width. Mice were randomly allocated to experimental groups, and the researcher performing tumor measurements was blinded to the experimental groups. All procedures were approved by the Bioethics Committee of Tsinghua University Shenzhen International Graduate School (Ethics Issue (2022) No. 94), and tumor sizes did not exceed the approved maximum dimensions or volumes.

### Statistical analysis

Data are presented as means ± SD or means ± SEM for three independent experiments, as specified. Statistical significance was assessed using two-tailed Student’s *t* tests or two-way ANOVA, as detailed in the figure legends. A p-value of less than 0.05 was considered statistically significant, with *p < 0.05, **p < 0.01, and ***p < 0.001 indicating increasing levels of significance.

## Results

### Metabolic profiling reveals a significant correlation between USP30 and glucose metabolism

**Fig. 1:**
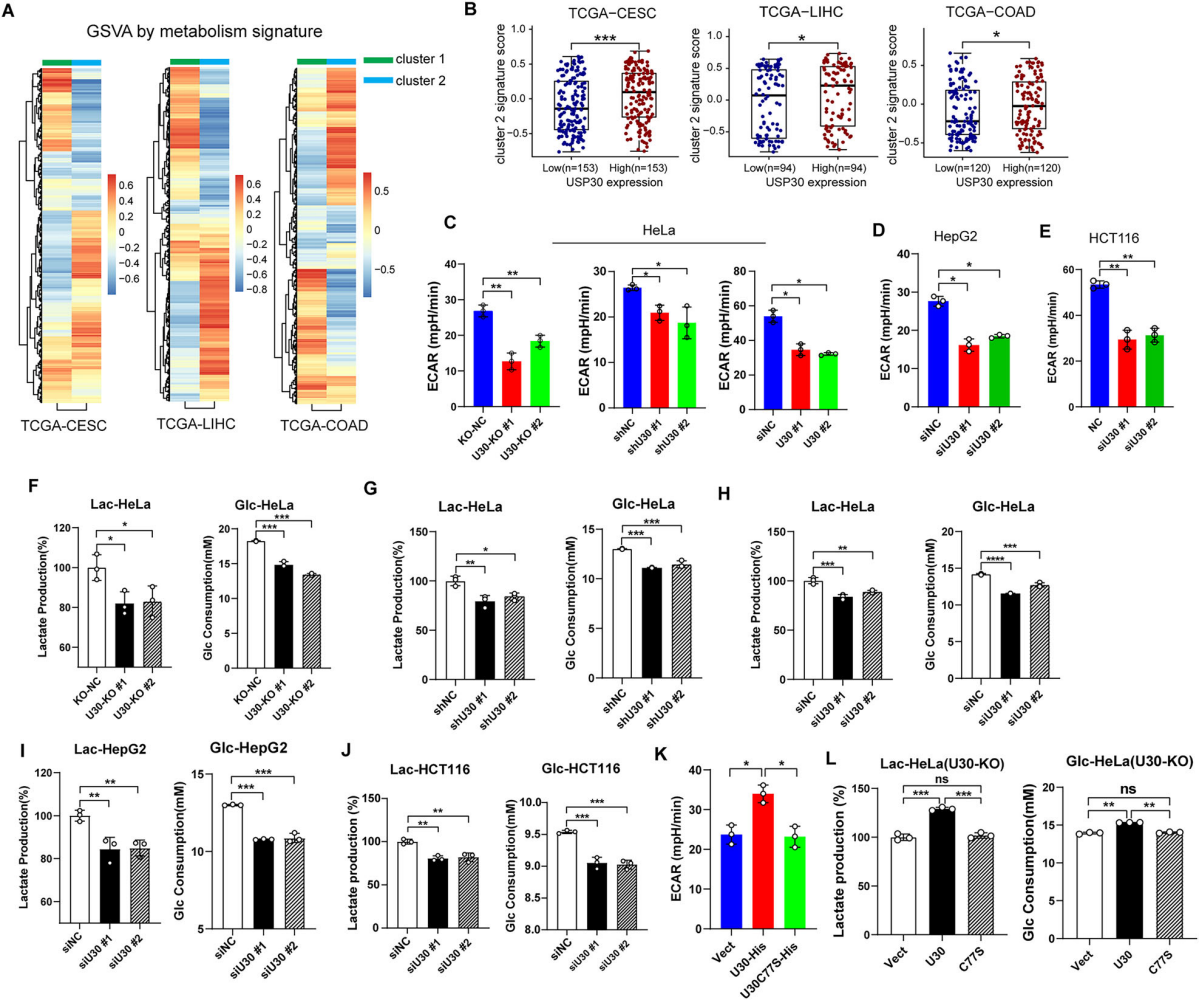
USP30 regulates tumor cell glucose metabolism. **A** Heatmap depicting the ssGSEA scores of two distinct pathways in tumor samples. Cluster1 corresponds to the “Lipid/Fatty Acid-Enriched” pathway, while Cluster 2 corresponds to the “Nucleotide/Carbohydrate-Enriched” pathway. Pathway scores were calculated using the ssGSEA algorithm applied to transcriptomic data from TCGA for cervical cancer (CESC), liver cancer (LIHC), and colorectal cancer (COAD). The magnitude of the score reflects pathway activity. **B** ssGSEA scores for the Cluster2 pathway in low- versus high-expression groups of *USP30*, normalized by USP30 expression levels. Extracellular acidification rate (ECAR) was measured in USP30-depleted HeLa (**C**), HepG2 (**D**) and HCT116 (**E**) cells using a Glycolytic Stress Test Kit. Lactate production and glucose consumption were quantified in USP30-depleted HeLa cells (**F**–**H**), HepG2 cells (**I**), and HCT116 cells (**J**). **K** Extracellular acidification rate (ECAR) was measured in HeLa cells expressing either USP30 WT or the C77S mutation. **L** Lactate production and glucose consumption in HeLa-KO cells expressing USP30 WT or C77S. Data are presented as mean ± SD of biologically independent samples (*n* ≥ 3). Statistical significance was determined by one-way ANOVA. ∗*p* < 0.05, ∗∗*p* < 0.01, and ∗∗∗*p* < 0.001.

Metabolic profiling of tumor samples with varying levels of USP30 expression provides valuable insights into the interplay between metabolic profiles and gene expression in cancer research. In breast cancer, metabolomics analyses have identified two main metabolic categories among tumor samples: one enriched in carbohydrates and nucleotides, and another characterized by elevated lipid and fatty acid levels [26]. Transcriptomic analysis has further linked these distinct metabolic profiles to specific gene expression signatures. To characterize these associations, we selected the top 200 genes associated with lipid metabolism (cluster 1) and glucose metabolism (cluster 2) to represent their respective pathways. We then applied the Gene Set Variation Analysis (GSVA) algorithm to transcriptomic data from cervical cancer (CESC), liver cancer (LIHC), and colon cancer (COAD) sourced from the TCGA (The Cancer Genome Atlas) database for pathway analysis. The resulting heatmap analysis revealed an inverse relationship between glucose and lipid metabolism activities across these cancer types. Specifically, tumor samples exhibiting heightened glucose metabolism tended to show reduced lipid metabolism, and vice versa (Fig. 1A).

To further investigate the role of USP30 in glucose metabolism, we stratified tumor samples based on USP30 expression levels into high and low expression groups. We focused our analysis on Cluster 2, which encompasses pathways associated with glucose metabolism, to assess their activity in these groups. As depicted in Fig. 1B, the high USP30 expression group demonstrated significantly elevated glucose metabolism activity compared to the low USP30 expression groups. This finding highlights the potential regulatory role of USP30 in modulating glucose metabolism pathways within the context of cancer.

### USP30 promotes glycolysis in tumor cells

To investigate the role of USP30 in regulating glucose metabolism in tumor cells, we employed gene knockout and knockdown approaches in HeLa cells and assessed their metabolic profiles using a Seahorse analyzer. Upon USP30 depletion, both the extracellular acidification rate (ECAR), indicating glycolytic activity, and the oxygen consumption rate (OCR), reflecting mitochondrial respiration, were markedly reduced (Fig. 1C, Supplementary Fig. S1A–E). These results suggested that USP30 deficiency diminishes both glycolytic flux and mitochondrial respiration capacity in HeLa cells. Previous studies have linked USP30 knockdown to increased mitophagy, which alters mitochondrial dynamics and compromises cellular respiration [15, 27, 28], consistent with our observed decrease in OCR after USP30 knockout.

Given the pivotal role of glycolysis in tumor progression, we further investigated the impact of USP30 on this metabolic pathway. We evaluated cellular glycolytic levels by measuring lactate production and glucose consumption in USP30 knockout or knockdown HeLa cells, revealing significant reductions in both parameters (Fig. 1F-H). To validate the broad relevance of these findings, similar experiments were conducted in HepG2 hepatocellular carcinoma and HCT116 colorectal cancer cells, both of which exhibited elevated USP30 expression levels [22]. Consistently, knockdown of USP30 led to decreased ECAR, glycolytic rate, lactate production, and glucose consumption in these cell lines (Fig. 1D, E, I, J, Supplementary Fig. S1F, G).

To ascertain whether USP30’s regulation of glycolysis involves its deubiquitinating enzyme activity, we overexpressed wild-type USP30 and a catalytically inactive mutant (C77S) in HeLa USP30-KO cells. Overexpression of wild-type USP30 significantly increased ECAR, lactate levels and glucose consumption compared to control cells transfected with an empty vector. In contrast, the catalytic-inactive mutant (C77S) did not elicit such effects (Fig. 1K, L, Supplementary Fig. S1H). These results demonstrate that USP30 promotes glycolysis in tumor cells through its deubiquitinating enzyme activity.

### USP30 interacts with multiple proteins involved in glycolysis

**Fig. 2:**
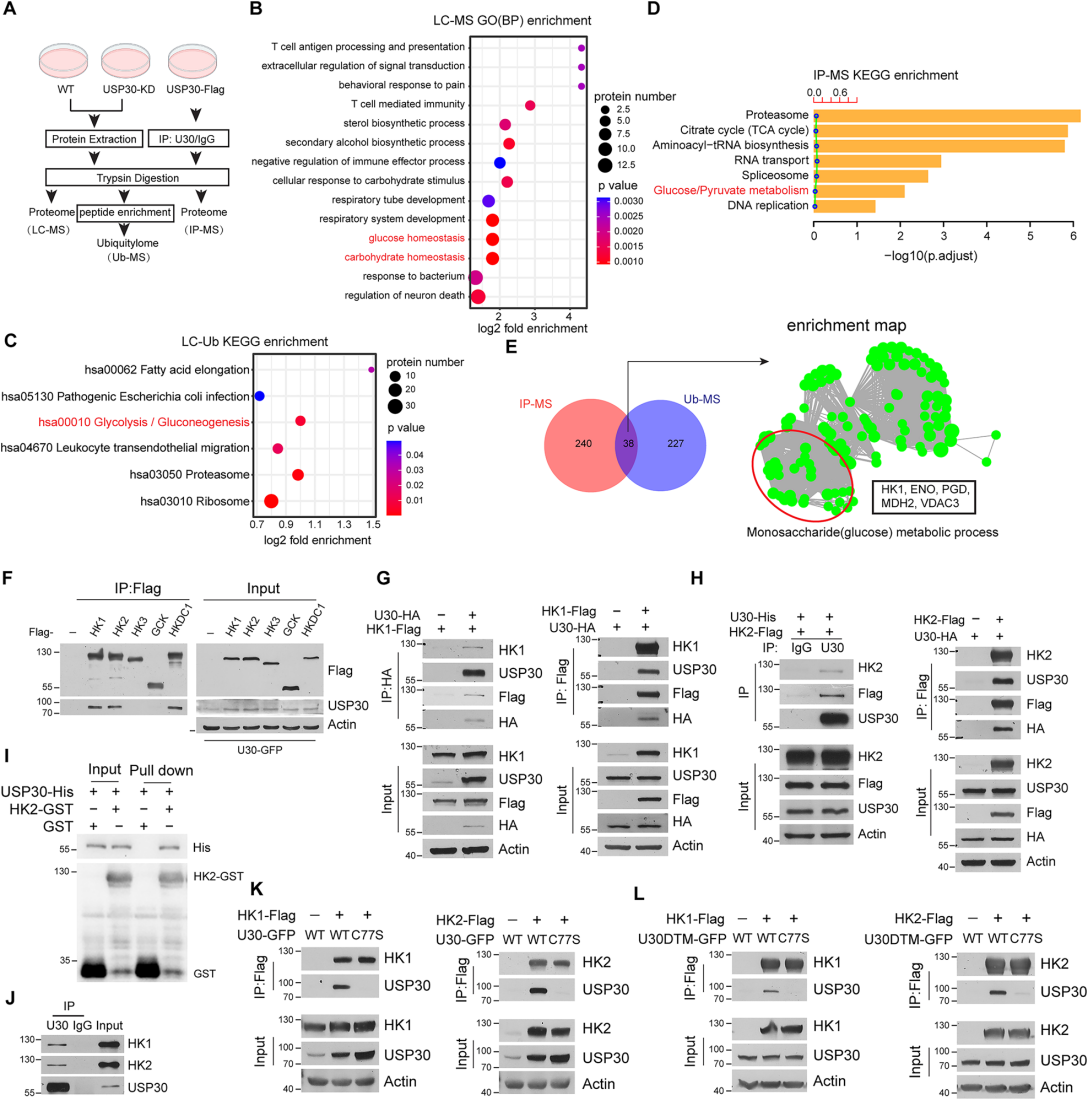
USP30 regulates glucose metabolism through interaction with hexokinases. **A** Quantitative proteomic and ubiquitinomics combined with IP-MS revealed USP30-dependent ubiquitination on target proteins. Enrichment analysis of proteins identified through LC-MS (**B**), Ub-MS (**C**), and IP-MS (**D**) in HeLa cells is presented. **E** Enrichment analysis of proteins co-enriched by IP-MS and Ub-MS revealed a series of kinases involved in carbohydrate metabolism. **F** Flag-tagged hexokinases (HK1, HK2, HK3, GCK, and HKDC1) and USP30-HA were co-expressed in HEK293 cells, followed by immunoprecipitation analyses using anti-Flag antibody. **G**, **H** HK1/HK2-Flag and USP30 were co-expressed in HEK293 cells, followed by immunoprecipitation of HA or Flag antibodies. **I** GST pull-down assay was performed by mixing purified GST-tagged HK2 protein with purified USP30-His. **J** Immunoprecipitation was performed on HeLa cell lysates using anti-USP30 or control IgG to analyze protein interactions. **K** HK1/HK2-Flag and USP30 (WT) and C77S mutation were expressed in HEK293 and immunoprecipitations of indicated proteins were performed. **L** HK1/HK2-Flag and USP30 with or without mitochondria transmembrane sequence, USP30-DTM WT and USP30-DTM C77S, were expressed in HEK293 and immunoprecipitations of indicated proteins were performed.

To elucidate the molecular mechanisms by which USP30 regulates glycolysis in tumor cells, we employed a comprehensive approach that combined USP30 immunoprecipitation coupled with mass spectrometry (IP-MS), quantitative proteomics (LC-MS), and ubiquitinomics (Ub-MS) technologies (Fig. 2A). Our analysis commenced with Gene Ontology (GO) enrichment of the differential proteomics data, filtered for fold change (FC > 1.5) and a significance threshold (*P* < 0.05). This analysis revealed several enriched signaling pathways influenced by USP30, including T cell antigen processing and presentation, extracellular regulation of signal transduction, T cell immunity, and notably, glucose and carbohydrate homeostasis (Fig. 2B). Furthermore, KEGG pathway enrichment analysis of the differential ubiquitinomic data from Ub-MS indicated significant enrichment in pathways such as fatty acid elongation, pathogenic *Escherichia coli* infection, and notably, glycolysis/gluconeogenesis (Fig. 2C). This enrichment encompassed numerous protein kinases associated with glycolysis, suggesting that USP30 may modulate these processes via ubiquitin modifications. Further investigation of proteins interacting with USP30 through IP-MS indicated an enrichment in pathways related to citrate cycle and glucose/pyruvate metabolism (Fig. 2D). Through integrated analysis of IP-MS and Ub-MS datasets, we identified a subset of proteins that not only interacted with USP30 but also exhibited altered ubiquitination levels. These proteins were notably enriched in pathways directly linked to glucose metabolism, featuring key glycolytic enzymes such as HK1, ENO, PGD, MDH2, and VDAC3 (Fig. 2E). These results implicate USP30 in the regulatory mechanisms of glycolysis through modulating the ubiquitination status of key glycolytic enzymes.

To validate this, we conducted immunoprecipitation experiments using a USP30 antibody, confirming its interaction with endogenous glycolytic proteins, including HK1, HK2, PFKP, and PFKM, all of which are critical rate-limiting enzymes in glycolysis (Supplementary Fig. S2A, B). Additionally, co-transfection experiments utilizing Flag-tagged glycolytic protein expression plasmids demonstrated specific interaction of USP30 with these glycolysis-related enzymes, reinforcing its role in the regulation of glycolytic pathways (Supplementary Fig. S2C, D).

### The interaction between USP30 and HK1/HK2

Hexokinases play a critical role in metabolism by catalyzing the phosphorylation of glucose, which contributes to the rate-limiting first step of glycolysis. The hexokinase family includes five members: HK1, HK2, HK3, GCK, and HKDC1. To investigate the interaction between hexokinases and USP30, we constructed expression plasmids for all five human hexokinases and co-transfected them with USP30-GFP for immunoprecipitation assays. Notably, HK1, HK2, and HKDC1 demonstrated significant interaction with USP30 (Fig. 2F). Subsequent analyses using TCGA pan-cancer RNA-Seq (https://portal.gdc.cancer.gov) and protein quantification from the Human Protein Atlas (HPA) database (https://proteinatlas.org) revealed that HK1 and HK2 exhibit markedly higher levels of gene expression and protein abundance across various tumor types compared with HKDC1, HK3, and GCK (Supplementary Fig. S3). Given these findings, we focused our investigation on HK1 and HK2. To further demonstrate the interaction between USP30 and HK1/HK2, we constructed Flag-tagged HK1 and HK2, co-transfected with USP30 expression constructs, and confirmed their interaction through co-immunoprecipitation with HA, Flag, or USP30 antibodies (Fig. 2G, H). We also conducted in vitro pull-down assays to validate the direct binding between USP30 and HK2 using recombinant proteins. His-USP30 efficiently bound GST-HK2, but not the GST-tag control (Fig. 2I, Supplementary Fig. S4A). Additionally, co-immunoprecipitation assay confirmed endogenous interaction between USP30 and both HK1 and HK2 (Fig. 2J). These results demonstrate that USP30-hexokinase binding occurs under physiological conditions and is not an artifact of overexpression systems.

To determine whether the deubiquitinase activity of USP30 is required for its binding to HK1 and HK2, we generated a catalytic inactive mutant (C77S). Co-immunoprecipitation experiments demonstrated that wild-type USP30 could bind to HK1 and HK2, whereas the catalytic inactive mutant failed to interact, indicating that this interaction is dependent on USP30’s enzymatic function (Fig. 2K). To determine whether USP30-HK1/HK2 complex formation requires the mitochondrial outer membrane (MOM) environment, we generated a transmembrane domain deletion mutant (USP30-DTM). As expected, USP30-DTM redistributed to the cytosol (Supplementary Fig. S4B, C). Notably, HK1 and HK2 retained interaction with this cytosolic USP30 mutant, suggesting that their interaction is independent of MOM localization. Crucially, a cytoplasmically localized catalytic inactive mutant (DTM-C77S) failed to co-immunoprecipitate with HK1 and HK2 (Fig. 2L). This dual mutation experiment establishes that USP30’s deubiquitinase activity, but not its mitochondrial tethering, is essential for hexokinase binding.

### HK1 and HK2 are novel deubiquitinating substrates of USP30

**Fig. 3:**
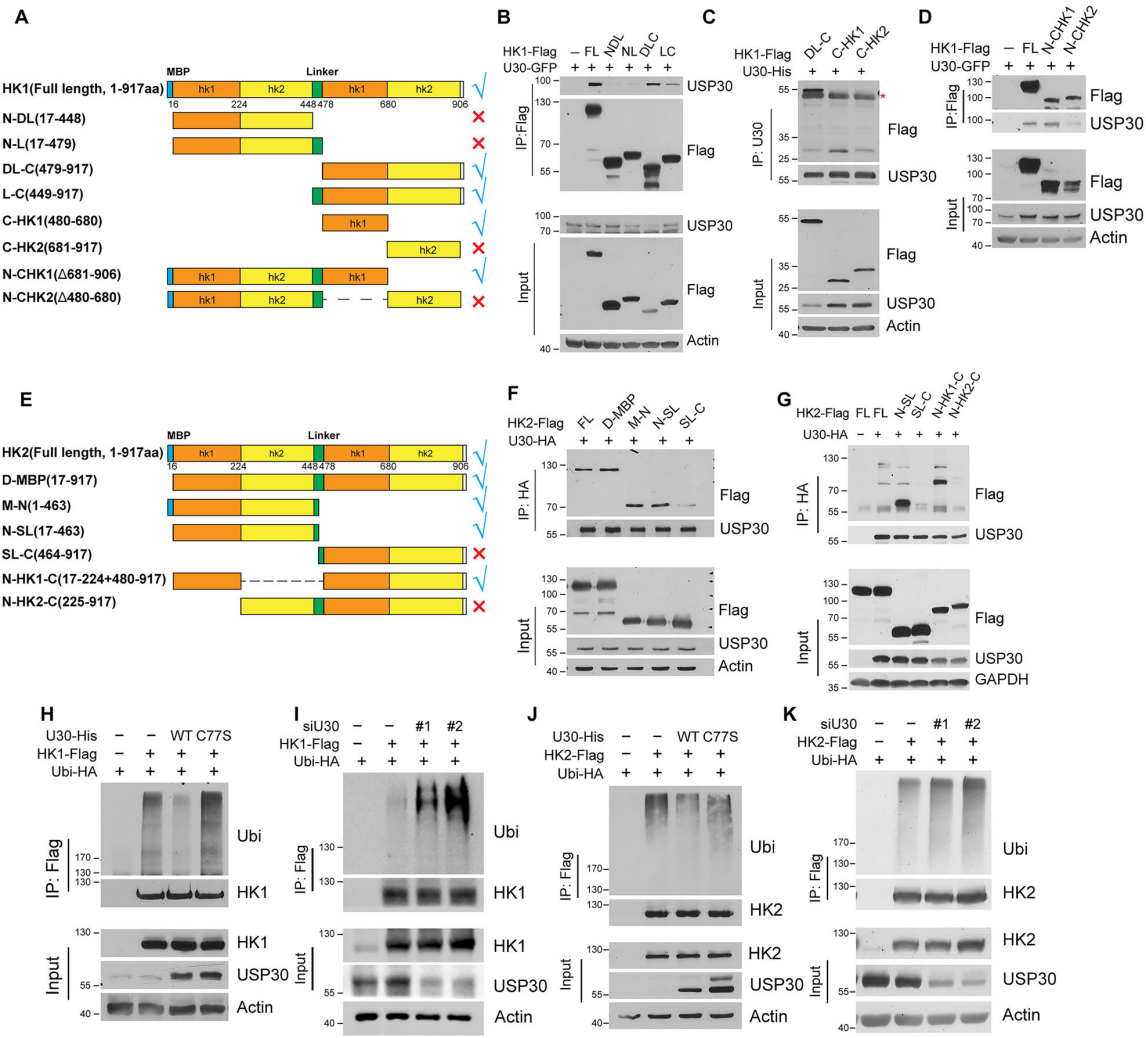
HK1 and HK2 are novel substrates of USP30 deubiquitinase. **A** Schematic representation of full length (FL) and truncated mutants of HK1. **B** USP30-GFP was co-expressed with Flag-tagged full length or truncated HK1 in HEK293 cells, followed by immunoprecipitation with Flag antibody. **C** The interaction between USP30 and truncated HK1 mutants, including DL-C, C-HK1, and C-HK2, was analyzed by co-immunoprecipitation with USP30 antibody. **D** Co-immunoprecipitation assay was performed to determine the interaction between USP30 and truncated HK1 mutants without the C-terminal sub-domain. **E** Schematic representation of full length (FL) and truncated mutants of HK2. **F**, **G** USP30-HA was co-transfected with Flag-tagged full length or truncated HK2 in HEK293 cells, followed by immunoprecipitation with HA antibody, the interaction between USP30 and HK2 mutants was analyzed by immunoblotting with indicated antibodies. **H, I** In vivo ubiquitination assay was performed to assess the ubiquitination level of HK1 in HEK293 cells transfected with USP30 plasmid or siRNA. **J, K** In vivo ubiquitination assay was performed to assess the ubiquitination leHvel of HK2 in HEK293 cells transfected with USP30 plasmid or siRNA.

Hexokinase 1 and 2 possess distinct N-terminal and C-terminal regions, which are connected by a linker of approximately 30 amino acids. Previous studies have suggested that this linker plays a regulatory role in the activity and stability of HK2 [29]. To investigate the functional significance of these regions, we engineered plasmids containing truncated mutations of HK1 and HK2, both with and without the linker (Fig. 3A, E). Co-immunoprecipitation analyses revealed that USP30 interacts specifically with the C-terminal region of HK1. Notably, the presence or absence of the linker did not significantly affect this interaction (Fig. 3B). Further dissection of the C-terminal domain identified a critical segment spanning amino acids 480–680 as essential for USP30 binding with HK1 (Fig. 3C, D).

In parallel, we examined the interaction domains between USP30 and HK2. Truncated fragments of HK2, derived from the full-length expression plasmid, were co-transfected with USP30-HA into HEK293 cells (Fig. 3E). Co-immunoprecipitation results demonstrated that USP30 could bind to all mutants containing the N-terminal region, highlighting its importance for USP30 interaction (Fig. 3F). Subsequent analysis identified a smaller N-terminal subdomain, specifically consisting of amino acids 16-224 as crucial for the USP30-HK2 interaction (Fig. 3G).

We further validated the role of USP30 in mediating deubiquitination of HK1 and HK2 through ubiquitination assays. The results showed that wild-type USP30 effectively reduced ubiquitination levels of HK1 and HK2, whereas the catalytically inactive mutant C77S failed to deubiquitinate these hexokinases (Fig. 3H, J). Conversely, knockdown of USP30 led to increased ubiquitination levels of HK1 and HK2 (Fig. 3I, K).

### USP30 stabilizes HK2 by removing atypical ubiquitin chains

**Fig. 4:**
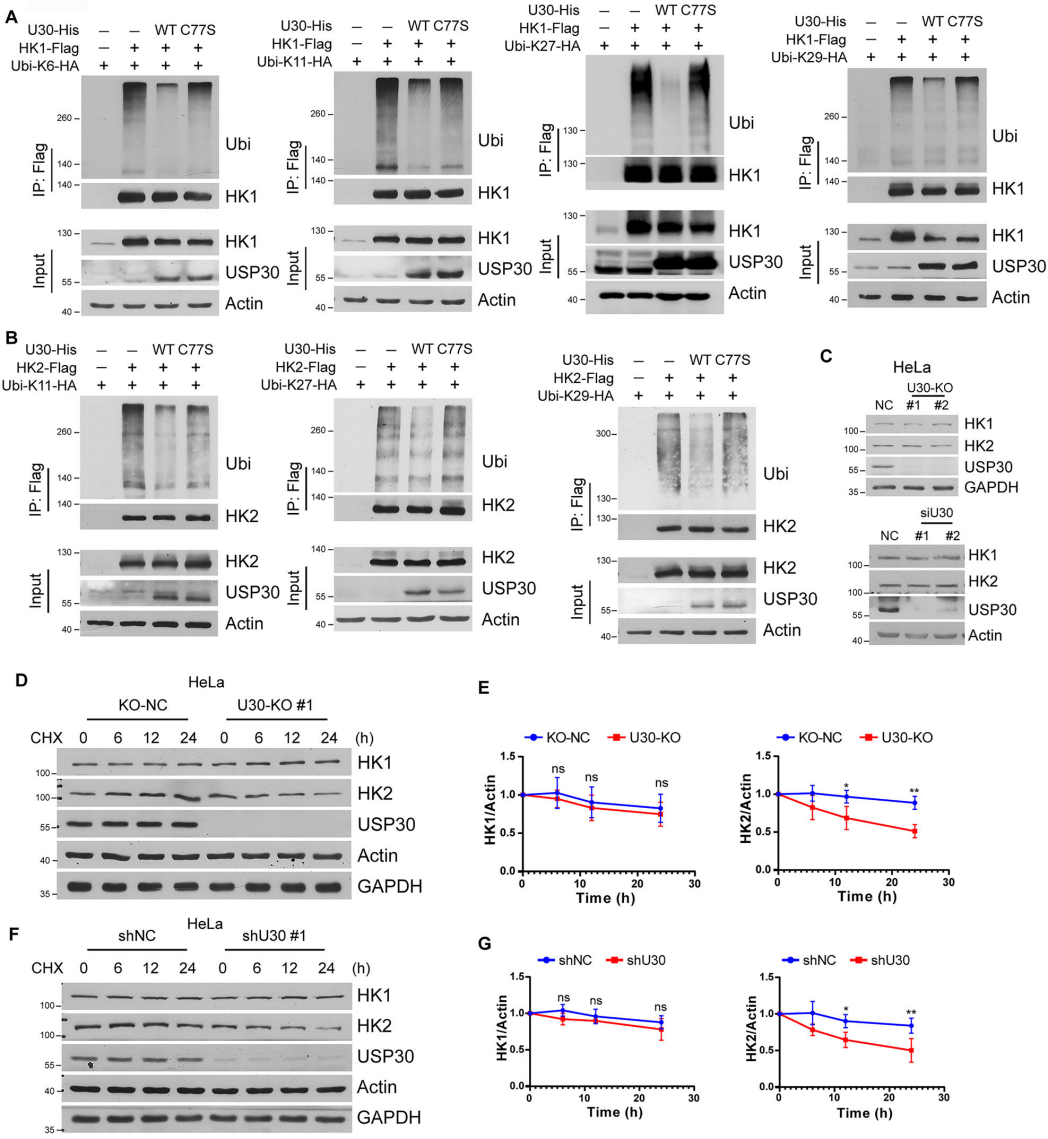
USP30 deubiquitinates atypical ubiquitin chains of HK1 and HK2 and regulates HK2 stability. **A, B** In vivo ubiquitination assays for HK1 and HK2 by USP30. HEK293 cells transfected with indicated plasmids were harvested for the ubiquitination assay using ubiquitin mutant plasmids. Cells were lysed with denaturing buffer, and ubiquitinated proteins were precipitated with anti-Flag beads before being analyzed by immunoblotting. **C** Western blotting analysis of HK1 and HK2 protein levels were performed in USP30-depleted HeLa cells. HeLa cells with USP30 knockout (**D**, **E**) or USP30 knockdown (**F**, **G**) were treated with cycloheximide (15 μg/mL) for the indicated periods. Cells were harvested for Western blotting. The intensities of the protein bands were quantified from three independent experiments (**E**, **G**), with the intensity of the 0-h time point set to 1 for comparison.

Ubiquitination modifications can be categorized by the type of ubiquitin chain linkage, including K6, K11, K27, K29, K33, K48, and K63. Previous studies have shown that USP30 predominantly removes K6 and K11-linked atypical ubiquitin chains from mitochondrial outer membrane proteins [18, 30]. To elucidate the role of USP30 in regulating HK1 and HK2, we co-transfected HEK293 cells with plasmids for mutated ubiquitin variants, either with either wild-type USP30 or the catalytically inactive C77S mutant, and either HK1 or HK2. For HK1, the ubiquitination assay demonstrated that USP30 primarily mediates deubiquitination of atypical ubiquitin chains, specifically K6-, K11-, K27-, and K29-linked chains (Fig. 4A; Supplementary Fig. S5B). In the case of HK2, USP30 predominantly removed K11, K27, and K29-linked polyubiquitin chains, with no significant impact on K6, K33, K48, and K63-linked chains (Fig. 4B; Supplementary Fig. S5A). These findings indicate that USP30 selectively targets atypical ubiquitin chains for deubiquitination of HK1 and HK2.

Ubiquitination plays a crucial role in regulating protein stability, subcellular localization, and enzymatic activity. Ubiquitin-mediated proteasomal degradation typically involves K48-linked ubiquitin chains. Our findings reveal that USP30 regulates atypical ubiquitin chains on HK1 and HK2, which primarily modulate protein function rather than stability. Knockout or knockdown of USP30 did not significantly alter total cellular levels of HK1 and HK2 in HeLa, HepG2, and HCT116 cells (Fig. 4C; Supplementary Fig. S5C–E). Next, we conducted cycloheximide (CHX) chase assays to analyze protein stability. HK1 stability was unaffected by USP30 depletion, whereas HK2 exhibited a significant reduction in protein half-life (Fig. 4D–G). These data establish that USP30-mediated deubiquitination preserves HK2 stability potentially through non-K48 chain removal.

### USP30 modulates the mitochondrial localization and kinase activity of HK2

**Fig. 5:**
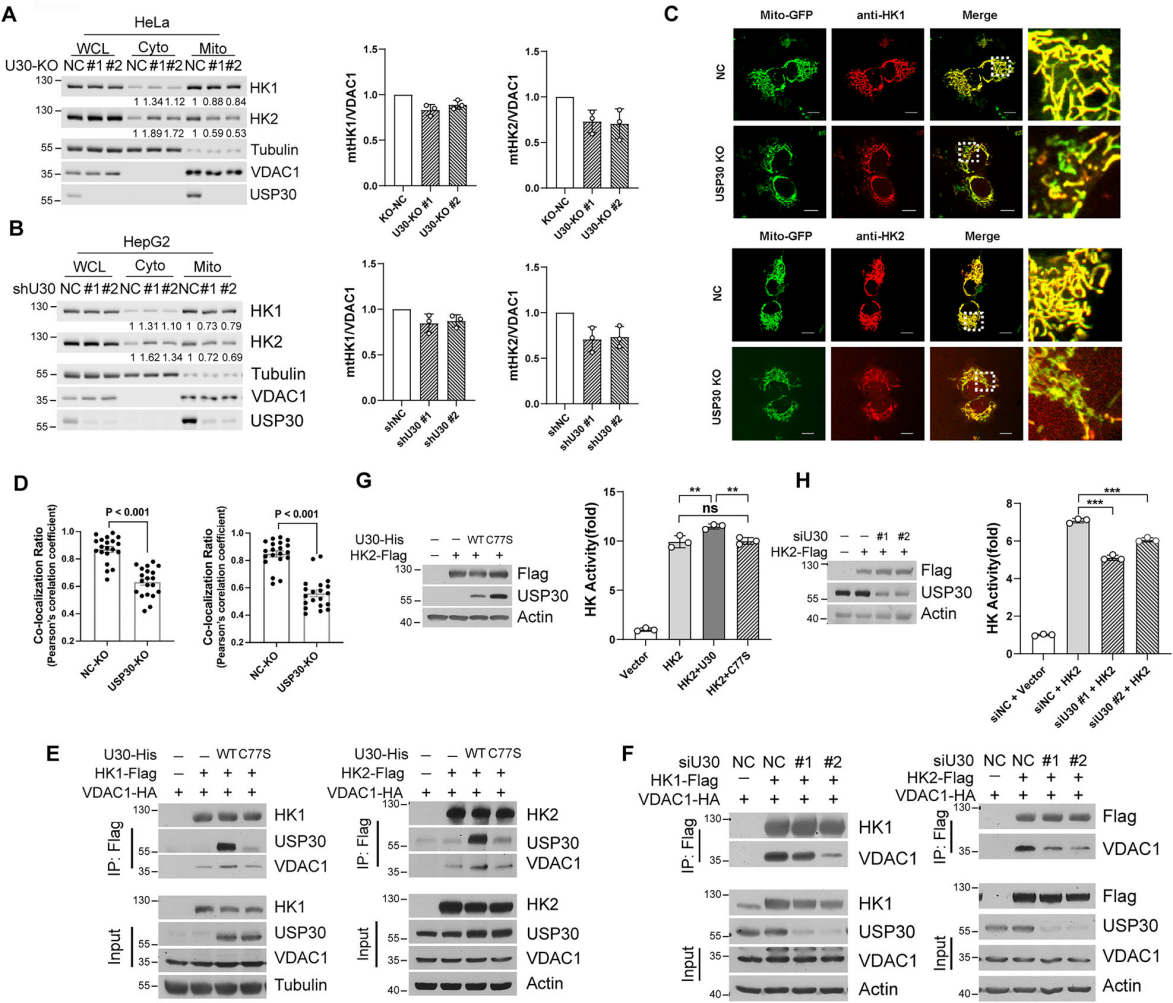
USP30 regulates HK1/HK2 subcellular localization and enzyme activity. **A** Western blot analyses of endogenous HK1 and HK2 in mitochondrial and cytoplasmic extracts from *USP30*-KO HeLa cells and **B** USP30 knockdown HepG2 cells. Quantification data from three experiments are shown (right panel). WCL, whole cell lysate; Cyto, cytoplasm; Mito, mitochondria. **C** *USP30*-KO HeLa cells transfected with Mito-GFP were fixed and subjected to immunofluorescence staining using indicated antibodies. Representative fluorescent images illustrate the co-localization of endogenous HK1 or HK2 (red) with mitochondria. Scale bar, 10 μm. **D** Quantification analyses of HK1/Mito-GFP and HK2/Mito-GFP co-localization in HeLa cells with USP30 knockout. The HK1/Mito-GFP and HK2/Mito-GFP correlation per cell was determined using the Pearson’s correlation coefficient. **E** Co-immunoprecipitation of Flag-tagged HK1 or HK2 with VDAC1 and USP30 in HEK293 cells transfected with the indicated plasmids. **F** HEK293 cells transfected with the indicated plasmids and *USP30* RNAi were harvested for co-immunoprecipitation. **G**, **H** HK2 kinase activity was evaluated in HEK293 cells transfected with the indicated plasmids.

HK1 and HK2 possess N-terminal mitochondrial targeting sequences that enable their association with the mitochondrial outer membrane, thereby enhancing glycolytic flux [31]. To test whether USP30 modulates glycolysis by affecting the mitochondrial localization of HK1 and HK2, we employed subcellular fractionation techniques in USP30 knockout (KO) HeLa and HepG2 cells. Immunoblotting analysis of the cytoplasmic and mitochondrial fractions revealed a significant redistribution of HK1 and HK2 upon USP30 depletion. Specifically, the levels of HK1 and HK2 increased in the cytoplasmic fraction, while their mitochondrial-bound forms decreased (Fig. 5A, B). To complement these findings, we performed immunofluorescence microscopy experiments. Compared with control cells, USP30-depleted cells exhibited a marked reduction in the co-localization of HK1 and HK2 with mitochondria (Fig. 5C, D).

When localized to the mitochondrial outer membrane, HK1 and HK2 interact with voltage-dependent anion channels (VDACs), which are critical for efficient transfer of ATP from mitochondria to the cytoplasm [7, 32]. To determine whether USP30 affects this interaction, we conducted co-immunoprecipitation assays in both whole cell extracts and isolated mitochondrial fractions. The results showed that wild-type USP30 enhanced the association between HK1/HK2 and VDAC1, whereas the catalytic inactive C77S mutant failed to promote this interaction (Fig. 5E, Supplementary Fig. S5F). Conversely, knockdown of endogenous USP30 led to a significant decrease in the binding of HK1/HK2 to VDAC1 (Fig. 5F; Supplementary Fig. S5G). These results suggest that USP30 regulates mitochondrial localization of HK1 and HK2, as well as their interaction with VDAC1, likely through deubiquitination-dependent mechanisms.

Notably, our results indicated that USP30 exerted a more pronounced effect on the protein stability and mitochondrial localization of HK2 compared to HK1. Therefore, we focused on elucidating the impact of USP30 on HK2 kinase activity. We co-expressed HK2 with either wild-type USP30 or C77S mutant in HEK293 cells and measured its kinase activity using a hexokinase activity assay kit. Overexpression of wild-type USP30 led to a significant increase in HK2 kinase activity, whereas the C77S mutant had no effect. In contrast, knockdown of endogenous USP30 resulted in a substantial decrease in HK2 kinase activity (Fig. 5G, H). These findings demonstrate that USP30-mediated deubiquitination plays a dual role in the regulation of HK2. By maintaining the mitochondrial localization of HK2 and enhancing its catalytic activity, USP30 synergistically promotes glycolytic rates, highlighting the importance of USP30-HK2 axis in cellular energy metabolism.

### USP30-mediated K144 deubiquitination stabilizes HK2 and promotes its mitochondrial targeting

**Fig. 6:**
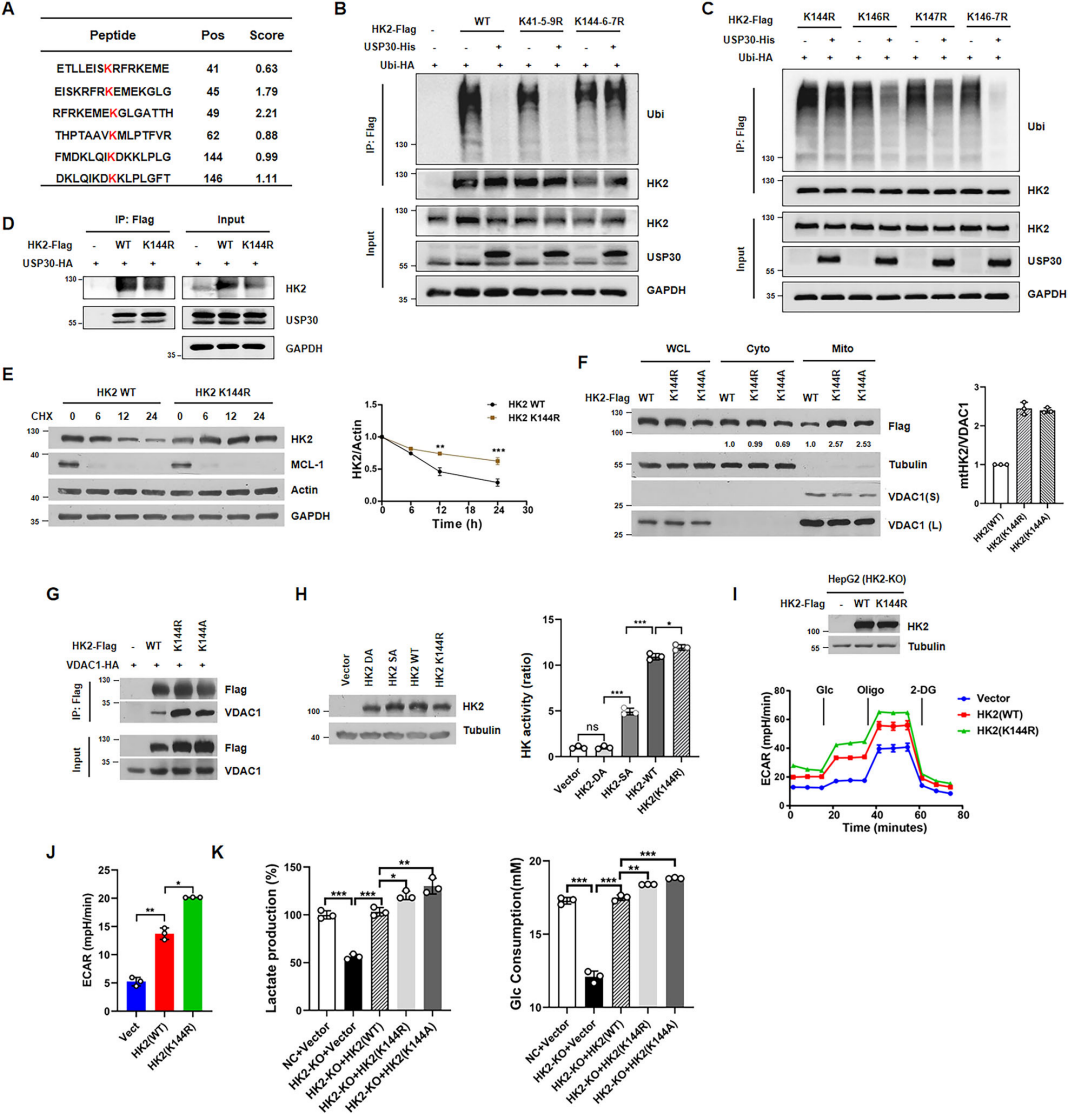
USP30 deubiquitinates HK2 at Lys144. **A** The predicted ubiquitination sites within the subdomain of the N-terminal region of HK2 are illustrated. **B** In vivo ubiquitination assays were conducted in HEK293 cells transfected with or without Ub-HA, alongside with HK2 wild-type (WT), K41R/K45R/K49R triple mutant (K41-5-9R), or K144R/146R/147R triple mutant (K144-6-7R). **C** In vivo ubiquitination assays in HEK293 cells transfected with or without Ub-HA, alongside with HK2 K144R, K146R, K147R, or K146R/147R double mutant (K146-7R). **D** HEK293 cells co-transfected with Flag-tagged HK2 WT or K144R and USP30-HA were lysed for immunoprecipitations, subsequently analyzed by Western blotting. **E** HEK293 cells transfected with HK2 WT or K144R were treated with CHX (20 µg/mL) and harvested at the indicated time points, followed by western blotting. The protein levels were quantified from three independent western blot analyses. **F** HEK293 cells expressing the specified mutant forms of HK2 were subjected to mitochondrial and cytoplasmic extraction, followed by western blotting using Flag and VDAC1 antibodies. The protein levels of mitochondrial bound HK2 were quantified from three independent western blot analyses. **G** Mitochondria purified from HEK293 cells transfected with the indicated plasmids were harvested for co-immunoprecipitation. The precipitated proteins were isolated with anti-Flag beads and analyzed by western blotting. **H** Hexokinase activity was measured for the indicated HK2 mutants. **I**, **J** Glycolytic activity was assessed in HepG2 cells with HK2 knockout (HK2-KO) rescued with HK2 WT or K144R. **K** Lactate production and glucose consumption were evaluated in HepG2 HK2-KO cells rescued with HK2 WT or K144 mutants.

Given that HK2 is preferentially expressed in tumor and exhibits a pronounced susceptibility to USP30 regulation, we focused on identifying USP30-targeted deubiquitination sites of HK2. Using the BDM-PUB predictor (http://bdmpub.biocuckoo.org/prediction.php), we prioritized lysine residues within HK2’s USP30-interacting N-terminal domain (Fig. 6A). We constructed Flag-tagged triple mutants K41R/K45R/K49R (K41-5-9R) and K144R/146R/147R (K144-6-7R) and assessed ubiquitination in USP30-expressing HEK293 cells. USP30 significantly reduced ubiquitination of both wild-type (WT) HK2 and the K41R/K45R/K49R triple mutant, but showed no activity toward the K144R/146 R/147 R mutant (Fig. 6B). This implicates K144, K146, or K147 as primary USP30 target sites. To further pinpoint critical lysine residues, we constructed expression vectors containing single (K144R, K146R, K147R) and double (K146R/147 R) mutants. Co-immunoprecipitation results revealed that USP30 significantly reduced the ubiquitination levels of K146R, K147R, and the K146R/147R double mutant, but failed to reduce ubiquitination of the K144R mutant (Fig. 6C). These results establish that K144 is the dominant USP30 deubiquitination site on HK2. Next, we performed Co-IP experiments comparing the interaction between USP30 and wild-type HK2 versus the ubiquitination-deficient mutant K144R. The results showed that USP30 co-immunoprecipitated with both HK2 WT and K144R, indicating that USP30’s catalytic domain is essential for recognizing ubiquitin chains on HK2, but not specifically at K144 (Fig. 6D).

Although USP30 depletion minimally affected total HK2 levels, CHX chase assays revealed that it regulated HK2 protein stability. Both HK2 WT and the K144R mutant degraded over time following CHX treatment, but the K144R mutant exhibited attenuated degradation, suggesting K144 ubiquitination accelerates turnover (Fig. 6E). Next, we examined K144’s role in subcellular distribution. Cell fractionation assay showed that K144 mutation increased mitochondrial HK2 levels around 2.5-fold (Fig. 6F). Co-immunoprecipitation experiments using isolated mitochondria confirmed enhanced VDAC1 binding in K144R mutants (Fig. 6G). These data demonstrate that USP30-mediated K144 deubiquitination promotes HK2 mitochondrial localization and enhances its interaction with VDAC1.

### Deubiquitination of HK2 at K144 enhances its kinase activity and tumor glycolysis

Given established links between lysine residues and HK2 regulation [29], we tested whether K144 ubiquitination status influences HK2 kinase activity. Two kinase inactive mutants of HK2, the catalytically dead mutant HK2-DA (D209/D657A) and the partially active mutant HK2-SA (S340/S788A), were used as experimental controls [11]. We overexpressed HK2 wild-type, DA, SA, and K144R mutants and assessed their enzymatic activity using a hexokinase assay kit. HK2-DA mutant showed no detectable kinase activity compared to the vector control, while the SA mutant exhibited reduced kinase activity relative to WT. Notably, the K144R mutant displayed significantly higher activity than WT, suggesting that the K144 deubiquitination intrinsically enhances HK2 kinase activity (Fig. 6H).

To elucidate the functional consequences of the K144 deubiquitination site in HK2, we overexpressed either wild-type HK2 or K144R mutant in HK2 knockout HepG2 and HeLa cells. Glycolytic flux assay showed that cells expressing the K144R mutant exhibited an elevated ECAR compared to those expressing wild-type HK2 (Fig. 6I–K; Supplementary Fig. S6A), indicating enhanced glycolytic flux. To further validate the physiological impact of the ubiquitination site mutation on HK2, we measured lactate production and glucose consumption in the culture medium. Consistently, cells expressing K144R mutant demonstrated a significant increase in both lactate production and glucose consumption compared to HK2 WT (Fig. 6L; Supplementary Fig. S6B, C). These results suggest that USP30-mediated deubiquitination at the K144 site of HK2 plays a pivotal role in regulating glycolytic activity in tumor cells

### USP30-mediated K144 deubiquitination of HK2 promotes tumor progression

**Fig. 7:**
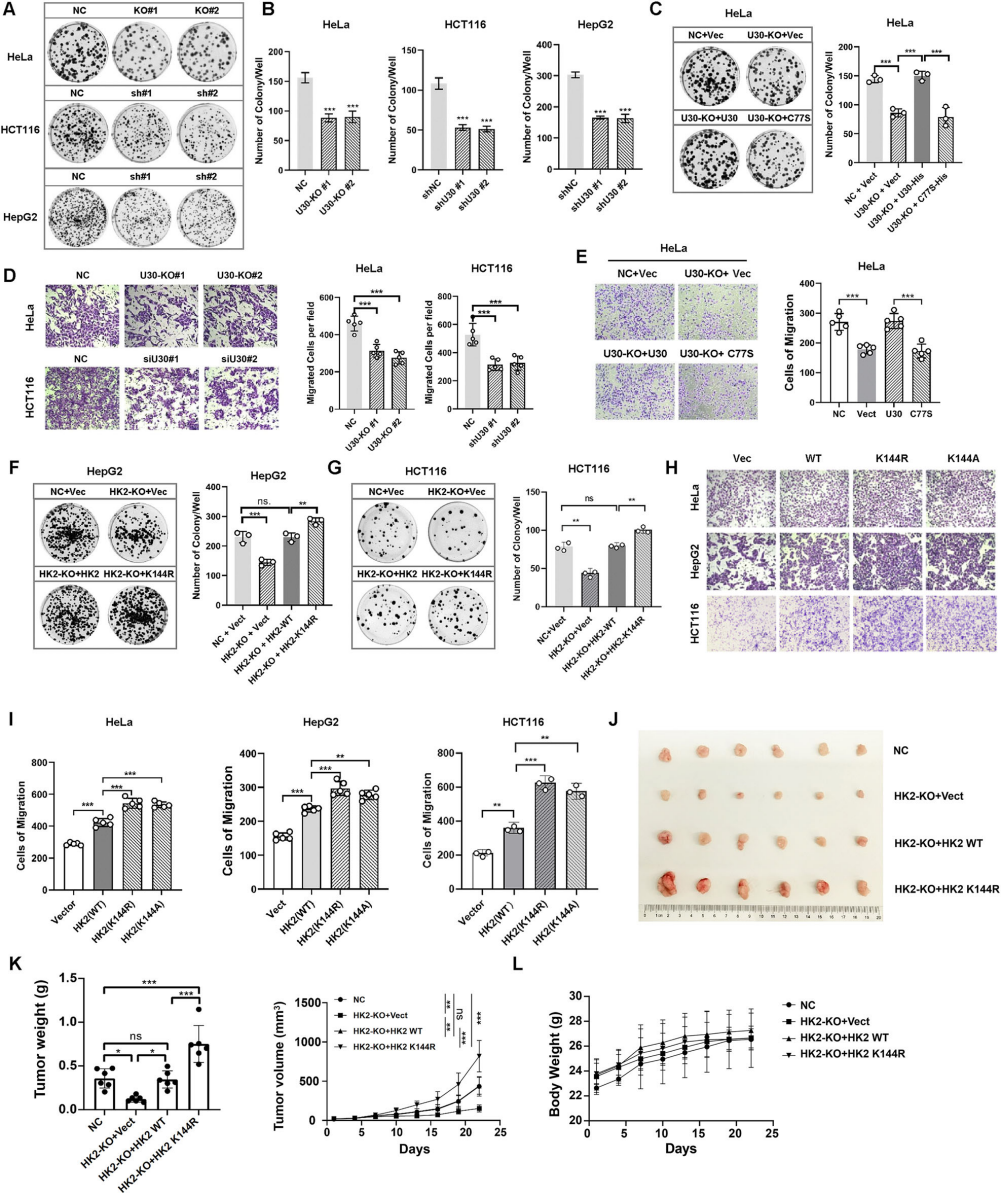
K144 ubiquitin-defective HK2 enhances tumor cell glycolysis, proliferation, and tumorigenesis. **A**, **B** Colony formation assays were conducted in HeLa, HepG2, and HCT116 cells following USP30 depletion, with quantification performed using Image J. **C** Colony formation assays in USP30 knockout (KO) HeLa cells reconstituted with USP30 WT or C77S mutant. The number of colonies was quantified using Image J. **D** Transwell migration assays were performed in HeLa and HCT116 cells with USP30 knockout or knockdown. **E** Transwell migration assays in HeLa USP30-KO cells reconstituted with USP30 wild-type or C77S mutant. **F**, **G** Colony formation assays in HepG2 and HCT116 HK2-KO cells rescued with HK2 WT or K144 mutants. **H** Transwell assays in HK2-KO HeLa and HepG2 cells rescued with HK2 WT or K144 mutants. **I**–**L** HK2-KO HepG2 cells with rescue expression of HK2 WT or K144R were subcutaneously injected into nude mice. Tumor weight and volumes (**K**) and body weight (**L**) were measured every three days. At the end of the experiment, tumors were photographed and weighted. Statistical significance was determined using one-way ANOVA followed by Tukey’s multiple comparisons test. Data are presented as mean ± SD.

To investigate the role of USP30 in tumor progression, we conducted cell proliferation and migration assays in HeLa, HepG2, and HCT116 cell lines. Genetic depletion of USP30 (KO) or shRNA knockdown significantly impaired tumor cell proliferation, colony formation, and migration (Fig. 7A, B, D; Supplementary Fig. S6D–F). Rescue experiments in HeLa USP30-KO cells revealed that wild-type USP30, but not the catalytically inactive C77S mutant, restored proliferation and migratory capacity (Fig. 7C, E; Supplementary Fig. S6G), demonstrating that USP30’s deubiquitinase activity is essential for these processes.

Next, we evaluated the functional impact of USP30-mediated deubiquitination at the K144 site of HK2. Reintroduction of wild-type HK2 or the K144 mutants into HK2-KO cells revealed that both constructs enhanced cell proliferation compared to vector controls. Notably, K144R and K144A-expressing cells exhibited significantly higher proliferation rates than the wild-type HK2 (Fig. 7F, G; Supplementary Fig. S6H–J). Similarly, transwell assays showed that K144R/A mutants promoted tumor cell migration more potently than HK2 WT (Fig. 7H, I), highlighting K144 as a critical residue for HK2-mediated metabolic reprogramming in cancer cells.

Finally, we employed a subcutaneous tumor xenograft model in nude mice. HK2-KO cells were stably transduced with lentiviral vectors expressing either wild-type HK2 or the K144R mutant. The transduced cells, along with negative control, were subcutaneously injected into separate groups of nude mice, and tumor growth was monitored every three days. Consistent with the in vitro findings, the HK2-KO group exhibited significantly reduced tumor volumes and weights compared to the negative control (NC) group. Reintroduction of wild-type HK2 restored tumor growth rates comparable to the NC group. Notably, however, tumors derived from cells expressing K144R mutant showed a marked increase in tumorigenic potential, characterized by accelerated growth rates and larger final tumor volumes and weights (Fig. 7J–L). These results provide conclusive evidence that the K144R mutation enhances glycolytic activity in tumor cells, thereby promoting cell proliferation and driving tumor growth in a physiological context.

## Discussion

PTMs of glycolytic pathway play crucial roles in regulating cancer development [33, 34]. While ubiquitination and ubiquitin-like modifications significantly influence cancer metabolic reprogramming, deubiquitinating enzymes (DUBs) directly regulating glycolytic enzymes remain less explored than E3 ubiquitin ligases [35]. This disparity highlights the need for a comprehensive study of deubiquitination within the context of cancer metabolism. In this study, we analyzed transcriptomic data from multiple cancer samples. Our findings revealed that samples with elevated USP30 expression exhibited pronounced glycolytic activity. Functional assays demonstrated that cells with USP30 knockout or knockdown exhibited reduced glycolytic activity. Through integrated LC-MS, Ub-MS, and IP-MS techniques, we identified that USP30 plays a pivotal role in regulating glycolytic activity in cancer cells by directly deubiquitinating key metabolic enzymes to regulate glycolysis. USP30 interacts with several glycolytic enzymes and modulates their ubiquitination status.

Hexokinase, the rate-limiting enzyme in the first step of glycolysis, is subjected to regulation by various E3 ubiquitin ligases and deubiquitinases. For instance, the E3 ligase TRAF6 catalyzes K63-linked polyubiquitination of HK2, which is crucial for its autophagic degradation and negative regulation of glycolysis [36]. In contrast, the E3 ligase HectH9 mediates K63-linked non-proteolytic ubiquitination of HK2, promoting glycolysis and facilitating the expansion of cancer stem cells [25]. CSN5 has been identified as a deubiquitinase that stabilizes HK2 expression, thereby enhancing glycolytic flux and promoting cancer metastasis in hepatocellular carcinoma [37]. Additionally, HK2 can undergo SUMOylation at lysine residues K315 and K492, and SENP1-mediated deSUMOylation facilitates HK2 mitochondrial localization and glycolytic activity [38]. The E3 ubiquitin ligase Parkin, which plays a role in regulating mitophagy, has also been shown to target HK1 for proteasomal degradation [39]. Here, we identify USP30 as a novel regulator that removes K6-, K11-, K27-, and K29-linked ubiquitin chains from both HK1 and HK2, indicating distinct regulation through atypical ubiquitin chain hydrolysis.

HK2 is the predominant isoform of hexokinases that is highly expressed in tumor tissues, playing a crucial role in sustaining elevated glycolytic flux in most cancer cells [40, 41]. In contrast, HK1 acts as a housekeeping gene, exhibiting widespread constitutive expression across various tissues and demonstrating a higher mitochondrial affinity than HK2 [32, 42]. Emerging evidence suggests HK1’s oncogenic role through interacting with KRAS4A, an isoform of the frequently mutated oncogene KRAS, during tumorigenesis [43, 44]. In this research, we demonstrate that USP30 binds to and deubiquitinates both HK1 and HK2 but targets distinct domains. Specifically, USP30 binds to the N-terminal small subunit (16-224 aa) of HK2 and the C-terminal small subunit (480–680 aa) of HK1. This distinction likely reflects the absence of kinase activity in the N-terminal region of HK1. Though USP30 maintains steady-state protein levels of HK1 and HK2, cycloheximide chase assays revealed that USP30 specifically affects HK2 stability. These data suggest that PTMs may impact HK2 half-life without altering basal expression.

In tumor cells, deletion of USP30 causes more pronounced HK2 dissociation from mitochondria than HK1. We identified lysine 144 (K144) as the critical USP30 deubiquitination site on HK2. This modification at the K144 residue plays a significant role in regulating HK2’s mitochondrial localization, VDAC1 interaction, and enzymatic activity. Notably, K144R mutation enhances glycolysis and accelerates tumor progression, underscoring the pivotal role of USP30 in modulating HK2 function and highlighting its potential as a therapeutic target in cancer metabolism. Previous studies have documented mutations at key lysine residues in HK2, such as K146 and K147, can influence enzyme activity, likely due to conformational changes that affect substrate binding and kinase activity [29]. In our study, we did not observe USP30-mediated deubiquitination at the lysine residues K146 and K147. Given K144’s location near the glucose-binding site, we propose that deubiquitination at this residue may stabilize an active HK2 conformation to enhance glucose binding and thereby affecting its kinase activity. Future structural validation is required to validate this hypothesis. Activity comparisons with catalytically impaired mutants (DA, D209/657A; SA, S340/788A) [11, 45] support the functional significance of K144R.

Collectively, this study establishes USP30 as a key regulator of cancer metabolism that modulates glycolytic flux and mitochondrial function. USP30 drives glycolysis by deubiquitinating HK2 at K144, removing atypical ubiquitin chains to enhance its mitochondrial localization, VDAC1 interaction, and kinase activity. By blocking HK2 ubiquitination, the K144R mutation phenocopies USP30 overexpression, resulting in elevated mitochondrial HK2 levels and activity, accelerating glycolysis and tumor progression, revealing an atypical ubiquitin-dependent mechanism controlling the Warburg effect. Furthermore, our findings demonstrate that increased glycolytic output fueled by USP30-HK2 signaling supplies key substrates and intermediates, thereby indirectly enhancing mitochondrial oxidative phosphorylation. Importantly, our IP-MS and Co-IP data reveal that USP30 also interacts with multiple TCA cycle enzymes, suggesting a potential broader role where USP30 may directly regulate TCA cycle activity and oxidative phosphorylation through the deubiquitination of key metabolic enzymes. These findings position USP30 as a central node integrating glycolytic flux and mitochondrial respiration through both direct enzyme regulation and substrate provision, uncovering novel layers of metabolic control in cancer.

## Supplementary information

Supplementary figures

original data

Table S1

## Data Availability

The data that support the findings of this study are available within the article and its supplementary information files. Additional data are available from the corresponding author upon reasonable request.
