# Correction: USP30-mediated Deubiquitination of Hexokinase 2 controls the metabolic fate of glucose and tumor progression

**DOI:** 10.1038/s41419-026-08841-8

**Published:** 2026-05-18

**Authors:** Zhang Haowei, Xiaolin Li, Weijie Liao, Xuan Qin, Yapei Jiang, Haitao Yang, Hongli Zeng, Yuetong Li, Weidong Xie, Yaou Zhang, Naihan Xu

**Affiliations:** 1 School of Food and Drug, Shenzhen Polytechnic University, Shenzhen, China; 2 State Key Laboratory of Chemical Oncogenomics, Institute of Biopharmaceutical and Health Engineering, Tsinghua Shenzhen International Graduate School, Tsinghua University, Shenzhen, China; 3 Department of Hematology and Oncology, International Cancer Center, Shenzhen University General Hospital, Shenzhen University, Shenzhen, China; 4 Fuwai Shenzhen Hospital, Chinese Academy of Medical Sciences, Shenzhen, China

**Keywords:** Cancer metabolism, Ubiquitylation, Ubiquitylation

Correction to: *Cell Death & Disease* 10.1038/s41419-026-08459-w, published online 14 February 2026

In the published article, two corrections have been made:

1. Figure 2I has been updated with the correct prote in band labels for the Western blot.
2. The ethics approval number previously cited as Et hics Issue (2022) No. 94 has been corrected to Ethics Issue (2021) No. 16.

All experimental data, results, and conclusions of the study remain unchanged and fully intact.

**original Figure 2** 
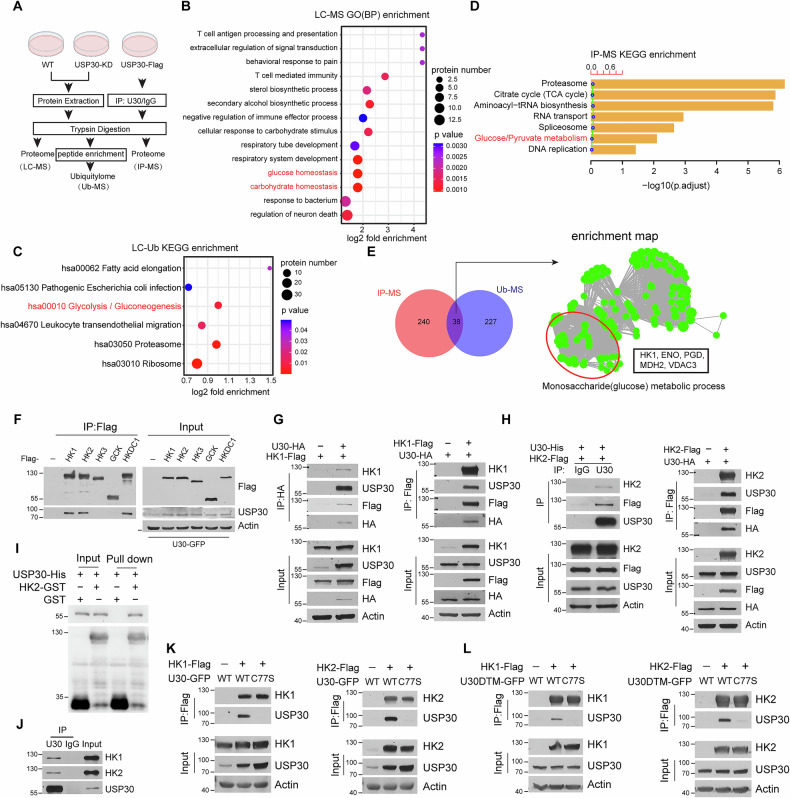


**corrected Figure 2** 
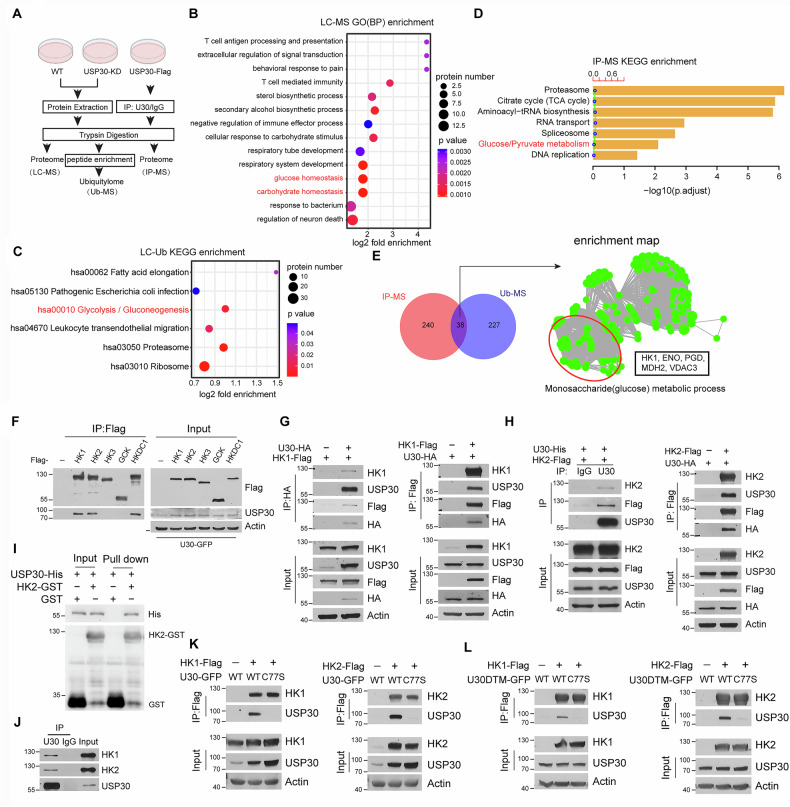


The original article has been corrected.

