# Supplementary material for: USP30-mediated Deubiquitination of Hexokinase 2 controls the metabolic fate of glucose and tumor progression
Source: Cell Death Dis. 2026 Feb 14;17(1):225. doi: 10.1038/s41419-026-08459-w (PMC12921045; doi:10.1038/s41419-026-08459-w)

**Supplementary Figures**

**Figure legends**

**Figure S1. USP30 regulates tumor cell glucose metabolism.**

1. Schematic representation of guide RNAs (gRNAs) designed to target *USP30* using the CRISPR/Cas9 system. Two gRNAs target exon2 and exon3, respectively. (B) Immunoblot analysis confirming the knockout of *USP30* in HeLa cell lines utilizing both gRNAs (left panel). Measurement of extracellular acidification rate (ECAR) in *USP30*-KO HeLa cells (right panel). (C) Oxygen consumption rate (OCR) was measured using *USP30*-KO HeLa cell lines. (D) Immunoblotting to verify the stable knock-down of *USP30* in HeLa cells using two shRNAs, followed by measurement of ECAR with the Glycolytic Stress Test kit. (E) Silencing of *USP30* in HeLa cells using two siRNAs, with subsequent measurement of ECAR. (F) Silencing of *USP30* in HepG2 and (G) HCT116 cells, followed by measurement of ECAR. (H) Re-expression of wild-type USP30 or C77S mutation in *USP30*-KO HeLa cells, followed by ECAR measurement. Statistical significance was determined by one-way ANOVA analysis. ∗p < 0.05, ∗∗p < 0.01, and ∗∗∗p < 0.001.

**Figure S2. USP30 regulates glucose metabolism through interaction with hexokinases.**

(A) Schematic representation of the glycolysis pathway. (B) Immunoprecipitation of HEK293 cell lysates was conducted using USP30 antibody or control IgG. (C) and (D) Flag-tagged isoforms of glycolytic enzymes and USP30-HA fusion proteins were expressed in HEK293 cells. Immunoprecipitation analyses were performed using anti-HA magnetic beads to examine protein-protein interactions.

**Figure S3. USP30 expression in pan cancer.**

1. Protein expression levels of the five hexokinase isoforms: HK1, HK2 (B), HK3 (C), GCK (D), and HKDC1 (E), analyzed through immunohistochemistry (IHC) data obtained from the Human Protein Atlas (HPA) across various cancer types. (F) Transcriptional expression levels of the same five hexokinase isoforms assessed using RNA sequencing (RNA-seq) data from The Cancer Genome Atlas (TCGA) across multiple cancer types.

**Figure S4. Pulldown assay and illustration of USP30 truncation and validation through immunoblotting.**

1. Ni-NTA pull-down assay was performed by mixing purified His-tagged USP30 protein with purified HK2-GST. (B) Schematic representation of USP30 truncations and mutations. (C) Western blot analysis of USP30 in subcellular fractions from HEK293 cells transfected with USP30 wild-type (WT) and a mutant variant with transmembrane deletion (DTM).

**Figure S5. Influence of USP30 on the deubiquitination and expression of HK1 and HK2.**

(A-B) In vivo ubiquitination assays demonstrating the effects of USP30 on HK2 (A) and HK1 (B). HEK293 cells were transfected with the indicated plasmids and harvested for ubiquitination assays using ubiquitin mutant plasmids. Cells were lysed and ubiquitinated proteins were precipitated using anti-Flag beads, followed by immunoblotting analysis. (C) Western blot analysis of HK1 and HK2 in HepG2 and (D) HCT116 cells with USP30 silencing by siRNA. (E) mRNA expression levels of HK1 and HK2 measured by quantitative PCR. (F) HEK293 cells transfected with the indicated plasmids and mitochondria were extracted, and the purified pellet were harvested with RIPA buffer for immunoprecipitation. The proteins of interest were precipitated with anti-Flag beads and subsequently analyzed by western blotting. (G) HEK293 cells transfected with the indicated plasmids and USP30 RNAi and mitochondria were harvested for immunoprecipitations. The indicated proteins were precipitated with anti-Flag beads and subjected to Western blotting.

**Figure S6. USP30 regulates tumor cell glycolysis and growth.**

(A) Expression of HK2 WT, K144R, and K144A in HK2 knockout (KO) HeLa cells, with subsequent measurement of extracellular acidification rate (ECAR). (B) Assessment of lactate production and (C) glucose consumption in HeLa cells with HK2 knockout (KO) and rescued expression of the corresponding HK2 mutants. (D) Cell proliferation assays conducted in HeLa, (E) HepG2, and (F) HCT116 cells following USP30 depletion. (G) Cell proliferation assays in USP30 knockout HeLa cells with rescued expression of USP30 WT and C77S mutant. (H) Cell proliferation assays in HK2 knockout HeLa, (I) HCT116 and (J) HepG2 cells with rescued expression of HK2 WT and K144 mutants.


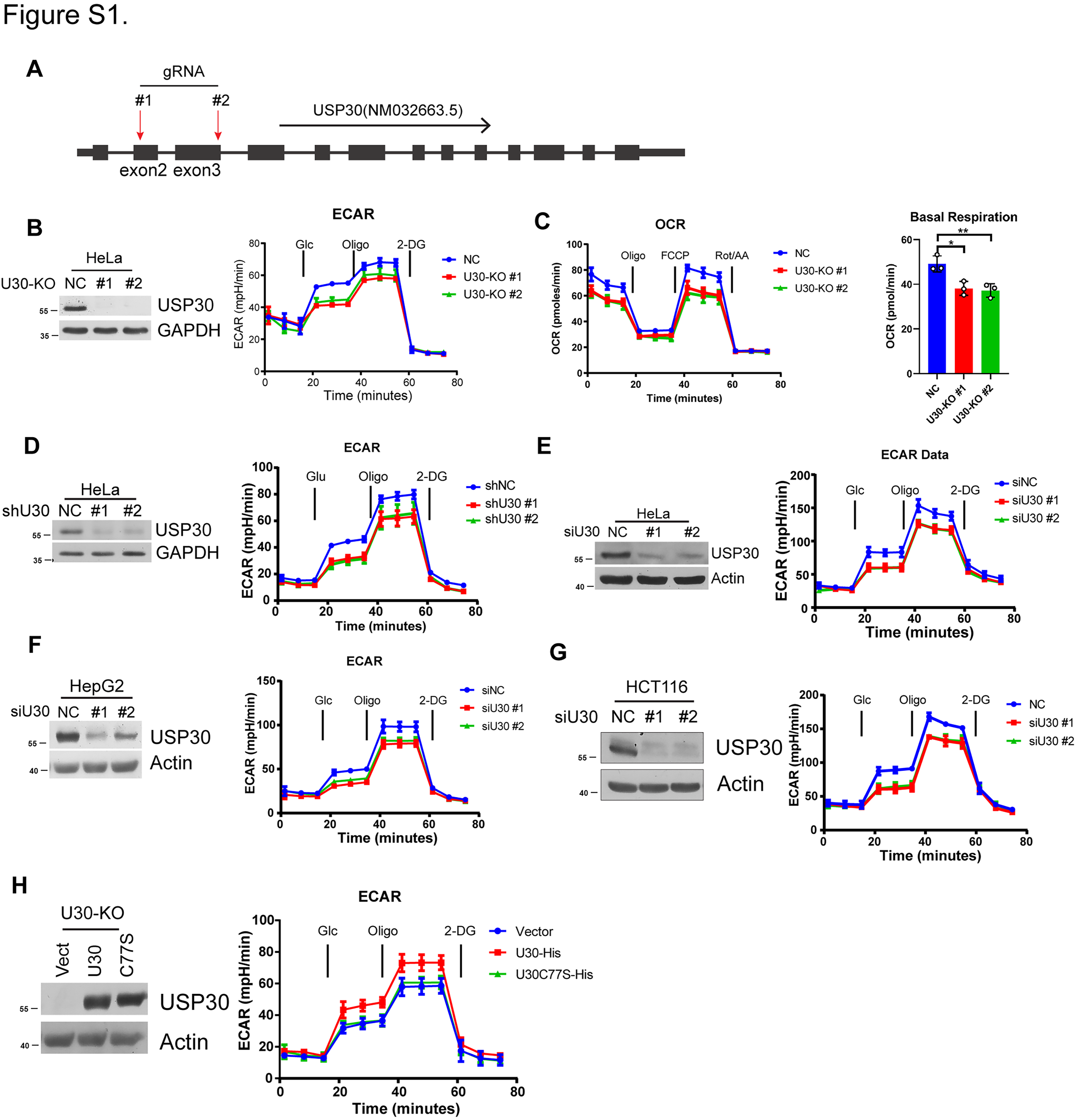


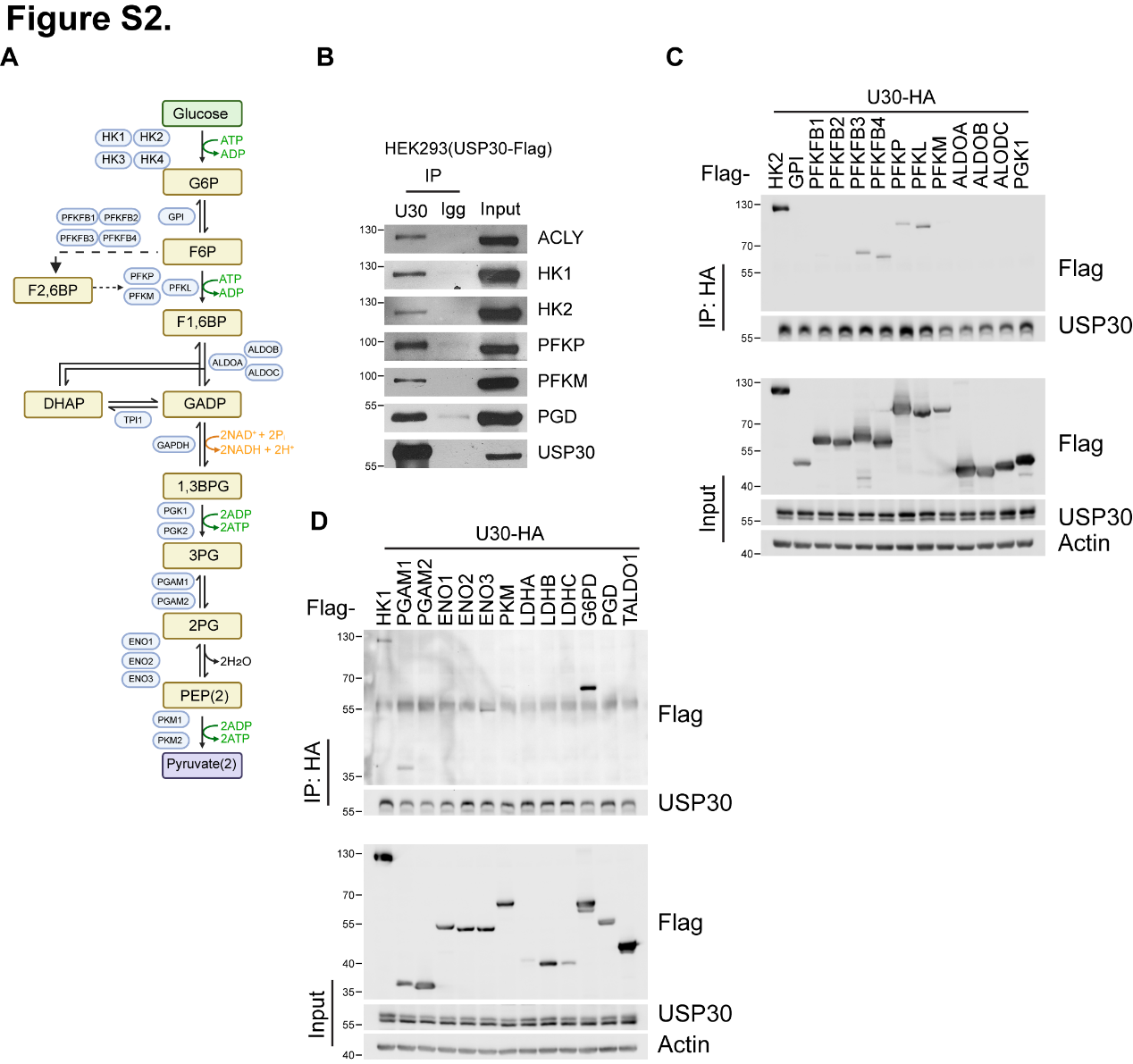


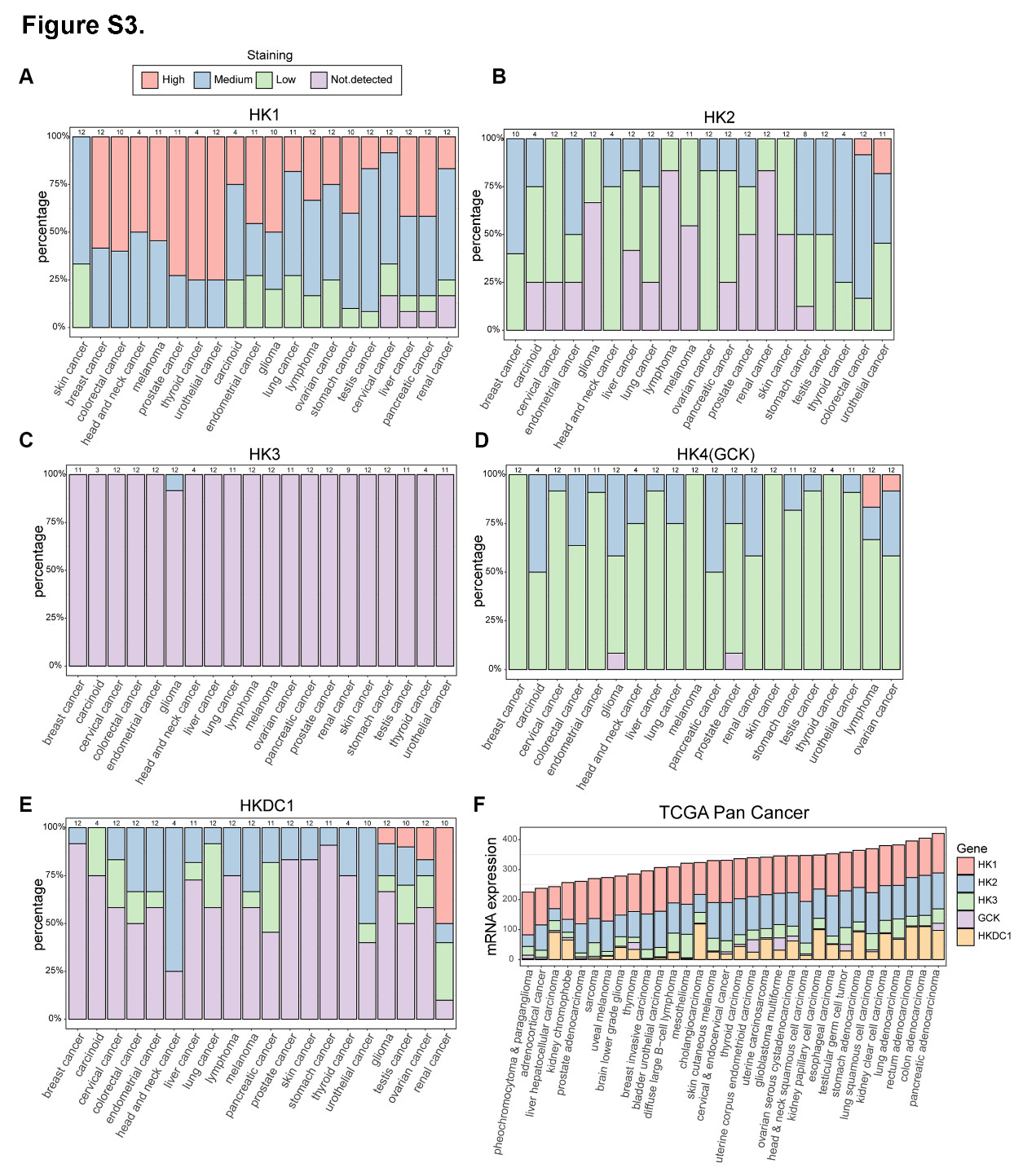


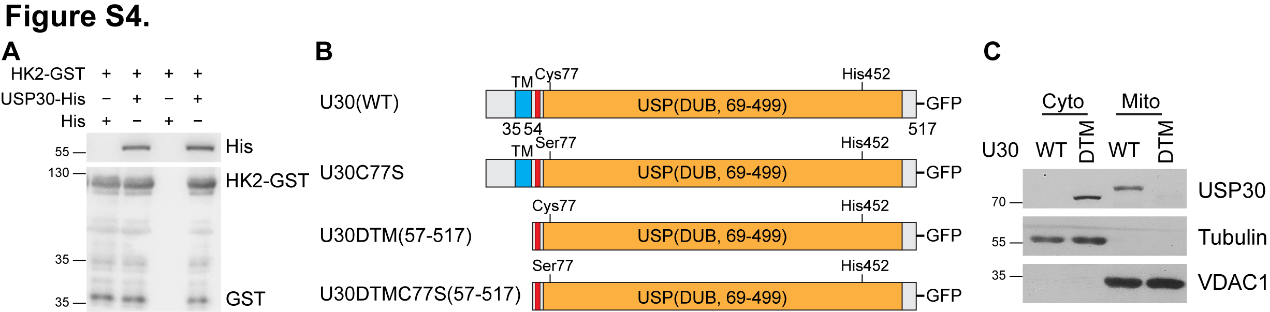


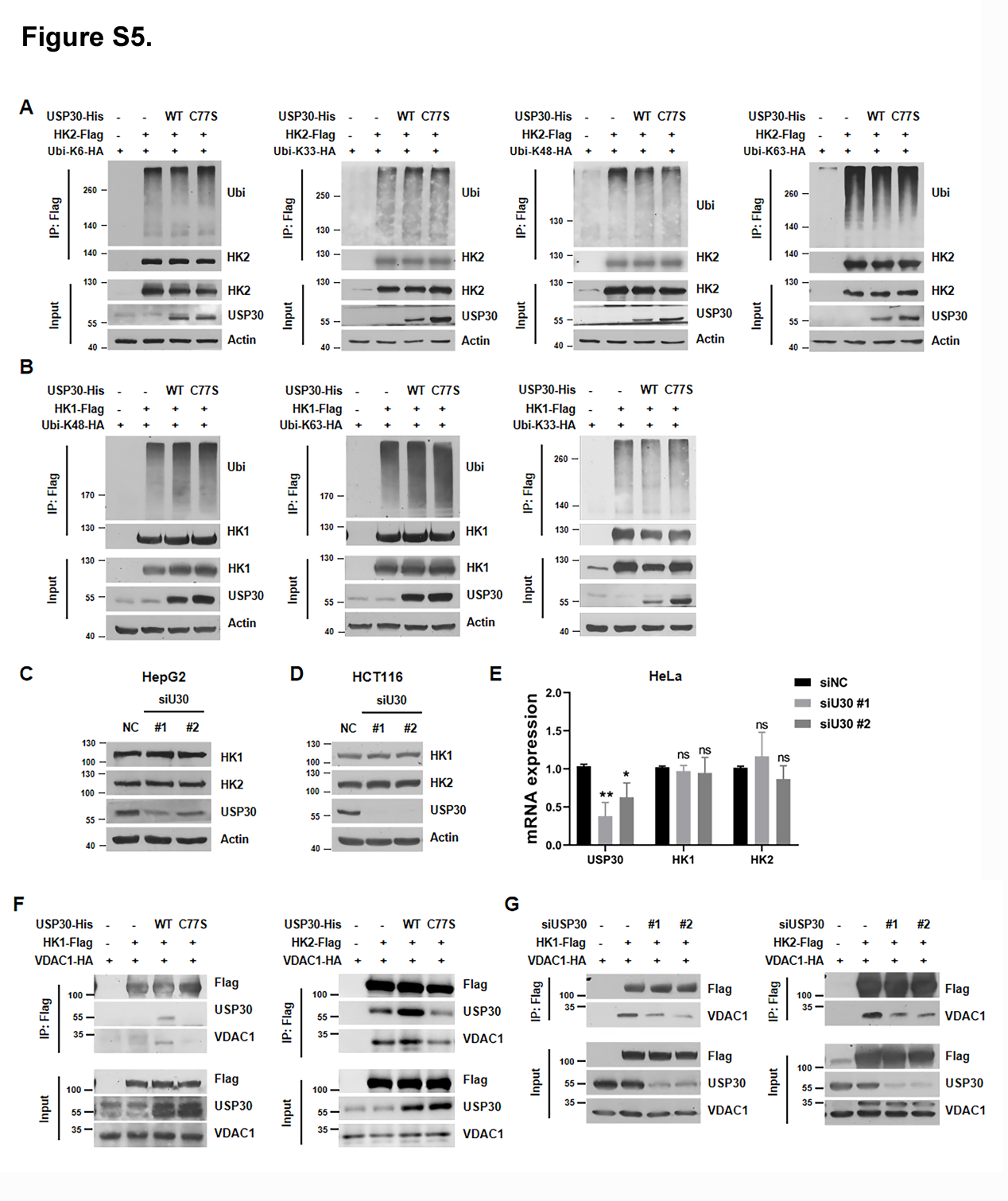


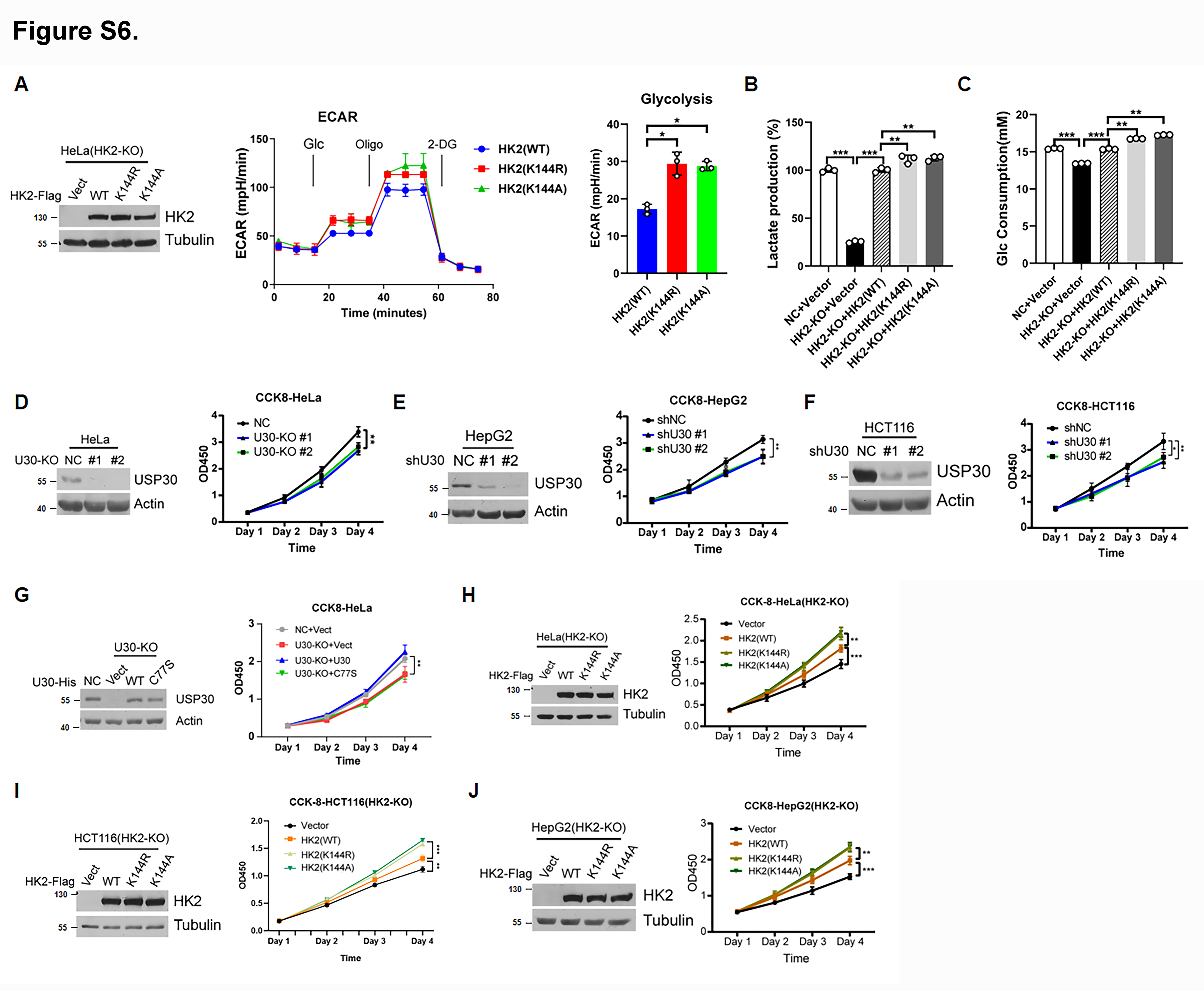

Supplement: Supplementary file 1 — Supplementary figures [file 41419_2026_8459_MOESM1_ESM.docx]
