# Supplementary material for: USP30-mediated Deubiquitination of Hexokinase 2 controls the metabolic fate of glucose and tumor progression
Source: Cell Death Dis. 2026 Feb 14;17(1):225. doi: 10.1038/s41419-026-08459-w (PMC12921045; doi:10.1038/s41419-026-08459-w)
Supplement: Supplementary file 3 — Table S1 [file 41419_2026_8459_MOESM3_ESM.docx]

**Table S1. Oligos for siRNAs, sgRNAs, and shRNAs**

| **siRNAs** |  |  |
| --- | --- | --- |
|  | **Forward Oligos (5’-3’)** | **Reverse Oligos (5’-3’)** |
| si*NC* | UUCUCCGAACGUGUCACGU | ACGUGACACGUUCGGAGAA |
| si*USP30* #1 | GCUGCUUGUUGGAUGUCUU | AAGACAUCCAACAAGCAGC |
| si*USP30* #2 | GGUUCUGUUGUGUUAAGAA | UUCUUAACACAACAGAACC |
|  |  |  |
| **sgRNAs for LentiCRISPRv2 and PX459 Vectors** | | |
|  | **Forward Oligos (5’-3’)** | **Reverse Oligos (5’-3’)** |
| *USP30* #1 | CACCGAAGAACTGGGGAGTTATAGG | AAACCCTATAACTCCCCAGTTCTTC |
| *USP30* #2 | CACCGAGTTCACCTCCCAGTACTCC | AAACGGAGTACTGGGAGGTGAACTC |
| *HK2* #1 | CACCGTTGTGAGGTCCACTCCAGAT | AAACATCTGGAGTGGACCTCACAAC |
| *HK2* #2 | CACCGTCAGATCTATGCCATCCCTG | AAACCAGGGATGGCATAGATCTGAC |
|  |  |  |
| **shRNAs for pLKO.1 Vector** | | |
|  | **Forward Oligos (5’-3’)** | **Reverse Oligos (5’-3’)** |
| sh*NC* | CCGGGCGCGATAGCGCTAATAATTTCTCGAGAAATTATTAGCGCTATCGCGCTTTTTG | AATTCAAAAAGCGCGATAGCGCTAATAATTTCTCGAGAAATTATTAGCGCTATCGCGC |
| sh*USP30* #1 | CCGGCCTAGTCAACACAACCCTAAACTCGAGTTTAGGGTTGTGTTGACTAGGTTTTTG | AATTCAAAAACCTAGTCAACACAACCCTAAACTCGAGTTTAGGGTTGTGTTGACTAGG |
| sh*USP30* #2 | CCGGGATAGCCTTTCACTAAGTATTCTCGAGAATACTTAGTGAAAGGCTATCTTTTTG | AATTCAAAAAGATAGCCTTTCACTAAGTATTCTCGAGAATACTTAGTGAAAGGCTATC |
|  |  |  |
