# Supplementary material for: USP30-mediated Deubiquitination of Hexokinase 2 controls the metabolic fate of glucose and tumor progression
Source: Cell Death Dis. 2026 Feb 14;17(1):225. doi: 10.1038/s41419-026-08459-w (PMC12921045; doi:10.1038/s41419-026-08459-w)
Supplement: Supplementary file 2 — original data [file 41419_2026_8459_MOESM2_ESM.docx]

Figure 2F

Figure 2G

Figure 2H

Figure 2I

Figure 2J

Figure 2K

Figure 2L

Figure 3B

Figure 3C

Figure 3D

Figure 3F

Figure 3G

Figure 3H

Figure 3I

Figure 3J

Figure 3K

Figure 4A1/2

Figure 4A3

Figure 4A4

Figure 4B1

Figure 4B2

Figure 4B3

Figure 4C

Figure 4D

Figure 4F

Figure 5A

Figure 5B

Figure 5G

Figure 5E1

Figure 5E2

Figure 5F

Figure 6B

Figure 6C

Figure 6D

Figure 6E

Figure 6F

Figure 6G

Figure 6H/I

Figure S1B/D/E/F/G/H

Figure S2B

Figure S2C

Figure S2D

Figure S4A

Figure S4C

Figure S5A

Figure S5B1/2

Figure S5B3

Figure S5C/D

Figure S5F1

Figure S5F2

Figure S5G1

Figure S5G2

Figure S6A/D/E/F/G/H/I

Figure S5E(Q-PCR)

|  |  | rep1 | rep2 | rep3 | rep4 |
| --- | --- | --- | --- | --- | --- |
| HeLa siNC |  |  |  |  |  |
|  | U30-II | 1.063223 | 1.038983 | 1.037806 | 1.000554 |
|  | HK1-1 | 1.043389 | 1.007549 | 1.012202 | 1.010823 |
|  | HK2-II | 1.021583 | 1.00007 | 1.04011 | 1.003848 |
|  |  |  |  |  |  |
|  |  |  |  |  |  |
| HeLa si*USP30* #1 |  |  |  |  |  |
|  | U30-II | 0.196078 | 0.199091 | 0.501007 | 0.370691 |
|  | HK1-1 | 0.732401 | 0.873963 | 0.77609 | 0.839044 |
|  | HK2-II | 1.107152 | 0.683514 | 1.257334 | 0.820636 |
|  |  |  |  |  |  |
|  |  |  |  |  |  |
| HeLa si*USP30* #2 |  |  |  |  |  |
|  | U30-II | 0.896355 | 0.47722 | 0.586151 | 0.5409 |
|  | HK1-1 | 0.724621 | 1.090102 | 0.8143 | 1.137367 |
|  | HK2-II | 0.956337 | 0.611199 | 0.982895 | 0.90638 |
|  |  |  |  |  |  |
